# Genetic Diversity of *HLA* Class I and Class II Alleles in Thai Populations: Contribution to Genotype-Guided Therapeutics

**DOI:** 10.3389/fphar.2020.00078

**Published:** 2020-02-27

**Authors:** Patompong Satapornpong, Pimonpan Jinda, Thawinee Jantararoungtong, Napatrupron Koomdee, Chonlawat Chaichan, Jirawat Pratoomwun, Chalitpon Na Nakorn, Wichai Aekplakorn, Alisa Wilantho, Chumpol Ngamphiw, Sissades Tongsima, Chonlaphat Sukasem

**Affiliations:** ^1^ Division of Pharmacogenomics and Personalized Medicine, Department of Pathology, Faculty of Medicine Ramathibodi Hospital, Mahidol University, Bangkok, Thailand; ^2^ Laboratory for Pharmacogenomics, Somdech Phra Debaratana Medical Center (SDMC), Ramathibodi Hospital, Bangkok, Thailand; ^3^ Division of General Pharmacy Practice, Department of Pharmaceutical Care, College of Pharmacy, Rangsit University, Pathum Thani, Thailand; ^4^ Graduate Program in Translational Medicine, Research Center, Faculty of Medicine Ramathibodi Hospital, Mahidol University, Bangkok, Thailand; ^5^ Department of Community Medicine, Faculty of Medicine Ramathibodi Hospital, Mahidol University, Bangkok, Thailand; ^6^ National Biobank of Thailand, National Science and Technology Development Agency, Pathum Thani, Thailand; ^7^ National Center for Genetic Engineering and Biotechnology, National Science and Technology Development Agency, Pathum Thani, Thailand; ^8^ The Thai Severe Cutaneous Adverse Drug Reaction (THAI-SCAR) Research Group, Bangkok, Thailand

**Keywords:** human leukocyte antigen, *HLA* class I, *HLA* class II, Thai population, pharmacogenetic marker

## Abstract

Human leukocyte antigen (HLA) class I and II are known to have association with severe cutaneous adverse reactions (SCARs) when exposing to certain drug treatment. Due to genetic differences at population level, drug hypersensitivity reactions are varied, and thus common pharmacogenetics markers for one country might be different from another country, for instance, HLA-A*31:01 is associated with carbamazepine (CBZ)-induced SCARs in European and Japanese while HLA-B*15:02 is associated with CBZ-induced Stevens–Johnson syndrome/toxic epidermal necrolysis (SJS/TEN) among Taiwanese and Southeast Asian. Such differences pose a major challenge to prevent drug hypersensitivity when pharmacogenetics cannot be ubiquitously and efficiently translated into clinic. Therefore, a population-wide study of the distribution of HLA-pharmacogenetics markers is needed. This work presents a study of Thai *HLA* alleles on both *HLA* class I and II genes from 470 unrelated Thai individuals by means of polymerase chain reaction sequence-specific oligonucleotide (PCR-SSO) in which oligonucleotide probes along the stretches of *HLA-A, -B, -C, -DRB1, -DQA1, and -DQB1* genes were genotyped. These 470 individuals were selected according to their regional locations, which were from North, Northeast, South, Central, and a capital city, Bangkok. Top ranked *HLA* alleles in Thai population include HLA-A*11:01 (26.06%), -B*46:01 (14.04%), -C* 01:02 (17.13%), -DRB1*12:02 (15.32%), -DQA1*01:01 (24.89%), and -DQB1*05:02 (21.28%). The results revealed that the distribution of HLA-pharmacogenetics alleles from the South had more HLA-B75 family that a typical HLA-B*15:02 pharmacogenetics test for SJS/TEN screening would not cover. Besides the view across the nation, when compared *HLA* alleles from Thai population with *HLA* alleles from both European and Asian countries, the distribution landscape of *HLA*-associated drug hypersensitivity across many countries could be observed. Consequently, this pharmacogenetics database offers a comprehensive view of pharmacogenetics marker distribution in Thailand that could be used as a reference for other Southeast Asian countries to validate the feasibility of their future pharmacogenetics deployment.

## Introduction

*Human leukocyte antigen (HLA)* gene is located on chromosome 6p21, which was considered the most polymorphic of human genetic system (Shiina et al., 2009). *HLA* encodes cell surface molecules that present antigenic peptides to the T-cell receptor (TCR) on T cells (Sette and Sidney, 1998). There are two main classes of HLA allele. *HLA* class I and II encode cell surface heterodimers that play a role in antigen presentation, tolerance, and self/nonself-recognition. *HLA* class I molecules gather peptides that have been synthesized within the individual nucleated cell, three main *HLA* class I genes including *HLA-A*, *HLA-B*, and *HLA-C* (Howell et al., 2010). Whereas *HLA* class II molecules gather exogenously synthesized peptide ligands by endocytic pathway and expressed with antigen-presenting cells (APCs), six main *HLA* class II genes including *HLA-DPA1*, *HLA-DPB1*, *HLA-DQA1*, *HLA-DQB1*, *HLA-DRA*, and *HLA-DRB1* (Ulvestad et al., 1994). The *HLA* system plays a critical role in regulating the immune response, tissue or organ transplantation, autoimmunity, vaccine development, susceptibility or resistance disease, and pharmacogenomics (Anania et al., 2017; Illing et al., 2017; Petersdorf, 2017). Over the past decade, there have been reported associations between various *HLA* alleles and different adverse drug reactions, especially severe cutaneous adverse reactions (SCARs).

One prominent report on drug-induced SCARs is the association between HLA-B*15:02 allele and carbamazepine (CBZ)-induced Stevens–Johnson syndrome/toxic epidermal necrolysis (SJS/TEN) in Han Chinese, Thai, and Southeast Asians (Ferrell and McLeod, 2008; Locharernkul et al., 2008; Hung et al., 2010; Chang et al., 2011; Nguyen et al., 2015). Conversely, Japanese and European populations were shown to have the association of HLA-A*31:01 and CBZ-induced hypersensitivity reactions (McCormack et al., 2011; Ozeki et al., 2011). While HLA-B*58:01 could be used as a pharmacogenetic risk prediction marker for allopurinol-induced SJS/TEN in many populations (Somkrua et al., 2011). In addition, the association between *HLA* class II and adverse drug reactions were reported, such as amoxicillin–clavulanate that induces liver injury was found to be associated with HLA-DRB1*15:01, HLA-DRB5*01:01, and HLA-DQB1*06:02 haplotype in European (Hautekeete et al., 1999; Lucena et al., 2011). Thus, the distributions of *HLA* alleles and pharmacogenetic markers that could vary among different populations might affect incidences of adverse drug reactions or drug dosage responses (Donnell PH and Dolan, 2009).

Although there is a clear need to investigate at a population level *HLA* alleles, a study on a distribution of Thai *HLA* alleles was limited. Puangpetch et al. (2015) previously reported only *HLA-B* polymorphisms from 986 Thai individuals. The top five of such *HLA-B* alleles consisted of HLA-B*46:01 (11.51%), HLA-B*58:01 (8.62%), HLA-B*40:01 (8.22%), HLA-B*15:02 (8.16%), and HLA-B*13:01 (6.95%). However, from this work, there were no reports of *HLA* class I and II allele in Thai population. There are other *HLA* alleles that play important roles in predicting various adverse drug reactions. Hence, the aim of this study was to comprehensively investigate both *HLA* class I (*HLA-A*, *-B*, and *-C*) and II (*HLA-DRB1*, *-DQA1*, and *-DQB1*) distribution of alleles in Thailand and the potential association with adverse drug reactions of these alleles.

## Materials and Methods

### Subjects

We recruited 470 unrelated healthy Thai individuals from the 4th National Health Examination Survey in Thailand during August 2008 and March 2009, and the information was obtained from National Health Examination Survey Office, Health System Research Institute, Ministry of Public Health, Thailand. The 470 Thai individuals were randomly chosen according to their *self-reported* origins which can be characterized into five regional groups: (n = 70) Bangkok, (n = 100) Central, (n = 100) Northeastern, (n = 100) Northern, and (n = 100) Southern. Since we want this study to represent majority of Thai people, subjects for each group must have lived in the aforementioned regions for more than three generations. Furthermore, these healthy individuals must have no history of cutaneous adverse drug reactions (CADRs). Thailand is a country located at the center of Southeast Asia, sharing boundaries with Myanmar (west), Laos (north east), Cambodia (east), and Malaysia (south). This study was approved by the Ethical Review Committee on Research Involving Human Subjects, Faculty of Medicine, Ramathibodi Hospital, Mahidol University. Written informed consent was obtained from all participants.

### *HLA* Class I and II Genotyping

Recruited genomic DNA samples were isolated from EDTA blood using the MagNAprue Compact Nucleic Acid Isolation kits (Roche Applied Science, Mannheim, Germany). The quality of genomic DNA was measured by NanoDrop^®^ ND-1000 (Thermo Scientific, Wilmington, USA). *HLA* class I alleles, comprising *HLA-A*, *HLA-B*, and *HLA-C*, and *HLA* class II alleles, comprising *HLA-DRB1*, *HLA-DQA1*, and *HLA-DQB1*, were genotyped using sequence-specific oligonucleotides (PCR-SSOs). Briefly, the DNA samples obtained from patients of the five regions in Thailand were amplified by polymerase chain reaction (PCR). The PCR products were hybridized against a panel of SSO probes on coated polystyrene microspheres that had sequences complementary to the stretches of polymorphism within the target *HLA* class I and II alleles using the Lifecodes HLA SSO typing kits (Immucor, West Avenue, Stamford, USA). The amplicon-probe complex was then visualized using a colorimetric reaction and fluorescence detection technology by the Luminex^®^IS 100 system (Luminex Corporation, Austin, Texas, USA). Interpretations of *HLA* class I and II alleles from the probe signals were performed using MATCH IT DNA software version 1.2.2 (One Lambda, Canoga Park, CA, USA).

### Statistical Analysis

The allele frequency and statistical analyses were performed using the Arlequin program version 3.1 for Hardy–Weinberg equilibrium testing. We used SPSS Compare allele frequencies between each region in Thailand using the SPSS software for Windows version 16.0 (SPSS Inc., Chicago, IL). A given pair of each region in Thai population was determined significant difference if the *p*-value was less than 0.05.

### Population Structure Analysis

There were only six *HLA* haplotypes from class I and II to be used in this population structure analysis which were not enough to investigate substructure from these 470 individuals. We employed *HLA* probe polymorphism signals obtained directly from each “stretch” of the six *HLA* haplotypes. The total number of probes used in this experiment was 403 polymorphism probes distributed across six *HLA* haplotypes. The numbers of probes for six *HLA* haplotypes are as follows: *HLA-A* (72 probes), *HLA-B* (92 probes), and *HLA-C* (77 probes) for *HLA* class I and *HLA-DRB1* (91 probes), *HLA-DQA1* (23 probes), and *HLA-DQB1* (48 probes) for *HLA* class II.

We concatenated the raw data containing probe signals for each *HLA*-haplotype into one tab-delimited file (Supplemental text: mergeallHLA.txt) containing a 470 403 HLA-probe matrix that was used in both principal component analysis (PCA) (Chaichompoo et al., 2018) and STRUCTURE analysis (Pritchard et al., 2000; Kopelman et al., 2015).

### Principal Component Analysis

Before performing PCA, the HLA-probe data entries were normalized based on z-score calculation. In particular, a probe signal *X* is converted to $X\prime =(X-\bar{X})/\mathrm{SD}$ where SD represents a standard deviation value of each probe column. The normalization step was done to minimize *HLA*-typing batch effects. We used *cal.pc.linear* function with default options from KRIS R package version 1.1.1 (Chaichompoo et al., 2018) to perform PCA. The PCA visualization was done using command *plot3views* from KRIS R package to display three main PCA perspectives, namely, PC1 vs. PC2, PC2 vs. PC3, and PC1 vs. PC3.

### STRUCTURE Analysis

The normalization of the raw probe data was done similar to PCA with an extra step to round all *X′* values into integer. The conversion was done so that STRUCTURE could treat these normalized signals as a type of variation patterns similar to that of microsatellites. For STRUCTURE analysis, we used 30,000 burnin length with 80,000 MCMC iterations. The analyses were done from K = 1 to K = 10 (K represents a number of Bayesian-inferred clusters in a given population) each of which had 30 repeats with the same parameter setting. The total 100 STRUCTURE analysis results were used as the input to CLUMPAK (Kopelman et al., 2015), which helped determine the optimal K based on an *ad hoc* quantity Delta (K) approach (Evanno et al., 2005). The STRUCTURE visualization was done using *pophelper* function from pophelper R package version 2.30 (Francis, 2017).

## Results

### *HLA* Class I and II Allele Frequencies in Thai Population

The allele frequency distribution of *HLA* class I and II are shown in **Tables 1**–**6**. We found that the alleles HLA-A*11:01, -A*24:02, -A*02:03, -A*33:03, -A*02:07, and -A*02:01 were more common than the others. HLA-A*11:01 was the most common allele found across five designated regions. For *HLA-B*, HLA-B*46:01 was the predominant allele commonly found in Northern, Northeastern, Central, and Bangkok regions. *HLA-B* allele profile from the Southern group, however, showed that HLA-B*15:02 was found more commonly (**Table 2**). Additionally, the allele distribution of *HLA-B* and their frequencies were HLA-B*46:01 (14.04%), -B*15:02 (7.66%), -B*40:01 (6.60%), -B*58:01 (6.38%), -B*13:01 (5.96%), -B*44:03 (4.47%), and -B*38:02 (4.26%), respectively. For *HLA-C,* the allele distribution and the corresponding frequencies were HLA-C*01:02 (17.13%), -C*07:02 (11.91%), -C*08:01 (10.32%), -C*03:04 (8.09%), -C*03:02 (7.77%), -C*07:01 (6.38%), and -C*07:04 (7.00%), respectively (**Table 3**). Particularly, HLA-C*08:01 (14.50%) allele was the highest frequency in the Southern group, but there was not significant difference when compared with the other regions (*p*-value = 0.4764) as shown in **Table 7**.

**Table 1:** The frequency of *HLA* class I alleles in Thai population and five regions (n = 470).

HLA-A	Thailand (n = 470)			Southern (n = 100)			Northern (n = 100)			Northeastern (n = 100)			Central (n = 100)			Bangkok (n = 70)		
	AF	CF	HW (p-value)	AF	CF	HW (p-value)	AF	CF	HW (p-value)	AF	CF	HW (p-value)	AF	CF	HW (p-value)	AF	CF	HW (p-value)
*01:01*	0.0223	0.04468	1.0000	0.0450	0.0900	1.0000	0.0050	0.0100	1.0000	0.0050	0.0100	1.0000	0.0250	0.0500	1.0000	0.0357	0.0714	1.0000
*01:03*	0.0011	0.00213	1.0000													0.0071	0.0143	1.0000
*02:01*	0.0553	0.10851	1.0000	0.0450	0.0800	1.0000	0.0400	0.0800	1.0000	0.0350	0.0700	1.0000	0.0700	0.1400	1.0000	0.1000	0.2000	1.0000
*02:02*	0.0011	0.00213	1.0000				0.0050	0.0100	1.0000									
*02:03*	0.1117	0.21064	1.0000	0.0400	0.0800	1.0000	0.1450	0.2800	1.0000	0.1400	0.2400	0.6827	0.1350	0.2600	1.0000	0.0929	0.1857	1.0000
*02:06*	0.0223	0.04468	1.0000	0.0200	0.0400	1.0000	0.0200	0.0400	1.0000	0.0300	0.0600	1.0000	0.0250	0.0500	1.0000	0.0143	0.0286	1.0000
*02:07*	0.0840	0.14468	0.0556	0.0500	0.0900	1.0000	0.1000	0.1700	1.0000	0.1000	0.1800	1.0000	0.0750	0.1300	1.0000	0.1000	0.1571	0.6193
*02:11*	0.0043	0.00851	1.0000	0.0100	0.0200	1.0000	0.0050	0.0100	1.0000	0.0050	0.0100	1.0000						
*02:17*	0.0043	0.00851	1.0000	0.0050	0.0100	1.0000							0.0100	0.0200	1.0000	0.0071	0.0143	1.0000
*02:20*	0.0011	0.00213	1.0000							0.0050	0.0100	1.0000						
*02:33*	0.0032	0.00426	1.0000							0.0050	0.0100	1.0000	0.0100	0.0100	1.0000			
*02:58*	0.0117	0.02340	1.0000	0.0050	0.0100	1.0000	0.0150	0.0300	1.0000				0.0250	0.0500	1.0000	0.0143	0.0286	1.0000
*03:01*	0.0064	0.01277	1.0000	0.0150	0.0300	1.0000				0.0050	0.0100	1.0000	0.0050	0.0100	1.0000	0.0071	0.0143	1.0000
*03:02*	0.0011	0.00213	1.0000							0.0050	0.0100	1.0000						
*11:01*	0.2606	0.44894	0.8984	0.2300	0.4100	1.0000	0.3550	0.5900	0.8307	0.2500	0.4600	0.7475	0.2600	0.4200	0.6162	0.1857	0.3286	1.0000
*11:02*	0.0181	0.03617	1.0000	0.0200	0.0400	1.0000	0.0300	0.0600	1.0000	0.0250	0.0500	1.0000	0.0050	0.0100	1.0000	0.0071	0.0143	1.0000
*11:03*	0.0032	0.00638	1.0000	0.0050	0.0100	1.0000							0.0050	0.0100	1.0000	0.0071	0.0143	1.0000
*11:04*	0.0053	0.01064	1.0000	0.0150	0.0300	1.0000				0.0050	0.0100	1.0000				0.0071	0.0143	1.0000
*11:09*	0.0011	0.00213	1.0000	0.0050	0.0100	1.0000												
*23:01*	0.0011	0.00213	1.0000	0.0050	0.0100	1.0000												
*24:01*	0.0011	0.00213	1.0000	0.0050	0.0100	1.0000												
*24:02*	0.1149	0.20213	0.1643	0.1450	0.2200	0.1679	0.1000	0.1900	1.0000	0.1150	0.2100	1.0000	0.1000	0.1900	1.0000	0.1143	0.2000	1.0000
*24:03*	0.0053	0.01064	1.0000							0.0100	0.0200	1.0000	0.0050	0.0100	1.0000	0.0143	0.0286	1.0000
*24:07*	0.0426	0.08298	1.0000	0.0700	0.1400	1.0000	0.0300	0.0600	1.0000	0.0250	0.0500	1.0000	0.0350	0.0700	1.0000	0.0571	0.1000	1.0000
*24:10*	0.0170	0.03404	1.0000	0.0100	0.0200	1.0000	0.0100	0.0200	1.0000	0.0400	0.0800	1.0000	0.0050	0.0100	1.0000	0.0214	0.0429	1.0000
*24:17*	0.0011	0.00213	1.0000	0.0050	0.0100	1.0000												
*24:25*	0.0011	0.00213	1.0000	0.0050	0.0100	1.0000												
*26:01*	0.0128	0.02553	1.0000	0.0150	0.0300	1.0000	0.0200	0.0400	1.0000	0.0050	0.0100	1.0000	0.0150	0.0300	1.0000	0.0071	0.0143	1.0000
*29:01*	0.0053	0.01064	1.0000				0.0050	0.0100	1.0000	0.0150	0.0300	1.0000	0.0050	0.0100	1.0000			
*29:10*	0.0011	0.00213	1.0000													0.0071	0.0143	1.0000
*30:01*	0.0213	0.04255	1.0000	0.0350	0.0700	1.0000	0.0100	0.0200	1.0000	0.0200	0.0400	1.0000	0.0250	0.0500	1.0000	0.0143	0.0286	1.0000
*31:01*	0.0085	0.01489	1.0000	0.0200	0.0300	1.0000	0.0050	0.0100	1.0000				0.0050	0.0100	1.0000	0.0143	0.0286	1.0000
*32:01*	0.0021	0.00426	1.0000	0.0050	0.0100	1.0000							0.0050	0.0100	1.0000			
*33:01*	0.0032	0.00638	1.0000				0.0050	0.0100	1.0000				0.0050	0.0100	1.0000	0.0071	0.0143	1.0000
*33:03*	0.1117	0.21064	1.0000	0.1050	0.1900	1.0000	0.0850	0.1700	1.0000	0.1000	0.1900	1.0000	0.1450	0.2600	1.0000	0.1286	0.2571	1.0000
*33:12*	0.0021	0.00426	1.0000	0.0100	0.0200	1.0000												
*34:01*	0.0096	0.01915	1.0000	0.0200	0.0400	1.0000	0.0050	0.0100	1.0000	0.0150	0.0300	1.0000				0.0071	0.0143	1.0000
*68:01*	0.0096	0.01915	1.0000	0.0200	0.0400	1.0000				0.0150	0.0300	1.0000				0.0143	0.0286	1.0000
*68:02*	0.0021	0.00426	1.0000	0.0100	0.0200	1.0000												
*74:01*	0.0064	0.01277	1.0000	0.0050	0.0100	1.0000				0.0200	0.0400	1.0000				0.0071	0.0143	1.0000
*74:05*	0.0021	0.00426	1.0000				0.0050	0.0100	1.0000	0.0050	0.0100	1.0000						

HLA-A, human leukocyte antigen-A; AF, allele frequency; CF, carrier frequency; HW, Hardy–Weinberg equilibrium; Significantly different p-value < 0.05.

**Table 2:** The frequency of *HLA* class I alleles in Thai population and five regions (n = 470).

HLA-B	Thailand (n = 470)			Southern (n = 100)			Northern (n = 100)			Northeastern (n = 100)			Central (n = 100)			Bangkok (n = 70)		
	AF	CF	HW (p-value)	AF	CF	HW (p-value)	AF	CF	HW (p-value)	AF	CF	HW (p-value)	AF	CF	HW (p-value)	AF	CF	HW (p-value)
*03:01*	0.0011	0.0021	1.0000	0.0050	0.0100	1.0000												
*03:02*	0.0011	0.0021	1.0000	0.0050	0.0100	1.0000												
*07:02*	0.0032	0.0064	1.0000	0.0100	0.0200	1.0000							0.0050	0.0100	1.0000			
*07:05*	0.0255	0.0511	1.0000	0.0050	0.0100	1.0000	0.0250	0.0500	1.0000	0.0400	0.0800	1.0000	0.0300	0.0600	1.0000	0.0286	0.0571	1.0000
*08:01*	0.0032	0.0064	1.0000	0.0050	0.0100	1.0000							0.0050	0.0100	1.0000	0.0071	0.0143	1.0000
*13:01*	0.0596	0.1149	1.0000	0.0350	0.0700	1.0000	0.0800	0.1400	0.4980	0.0600	0.1200	1.0000	0.0650	0.1300	1.0000	0.0571	0.1143	1.0000
*13:02*	0.0213	0.0426	1.0000	0.0350	0.0700	1.0000	0.0150	0.0300	1.0000	0.0150	0.0300	1.0000	0.0150	0.0300	1.0000	0.0286	0.0571	1.0000
*13:10*	0.0011	0.0021	1.0000				0.0050	0.0100	1.0000									
*14:02*	0.0011	0.0021	1.0000													0.0071	0.0143	1.0000
*15:01*	0.0053	0.0106	1.0000	0.0050	0.0100	1.0000	0.0100	0.0200	1.0000	0.0100	0.0200	1.0000						
*15:02*	0.0766	0.1511	1.0000	0.0900	0.1800	1.0000	0.0650	0.1300	1.0000	0.0650	0.1200	1.0000	0.0750	0.1500	1.0000	0.0929	0.1857	1.0000
*15:04*	0.0011	0.0021	1.0000	0.0050	0.0100	1.0000												
*15:07*	0.0021	0.0043	1.0000				0.0100	0.0200	1.0000									
*15:10*	0.0021	0.0043	1.0000	0.0050	0.0100	1.0000				0.0050	0.0100	1.0000						
*15:11*	0.0021	0.0043	1.0000	0.0050	0.0100	1.0000	0.0050	0.0100	1.0000									
*15:12*	0.0021	0.0043	1.0000				0.0050	0.0100	1.0000	0.0050	0.0100	1.0000						
*15:13*	0.0096	0.0191	1.0000	0.0250	0.0500	1.0000				0.0050	0.0100	1.0000	0.0100	0.0200	1.0000	0.0071	0.0143	1.0000
*15:17*	0.0043	0.0085	1.0000	0.0100	0.0200	1.0000							0.0050	0.0100	1.0000	0.0071	0.0143	1.0000
*15:18*	0.0032	0.0064	1.0000	0.0050	0.0100	1.0000										0.0143	0.0286	1.0000
*15:20*	0.0011	0.0021	1.0000				0.0050	0.0100	1.0000									
*15:21*	0.0021	0.0043	1.0000	0.0050	0.0100	1.0000				0.0050	0.0100	1.0000						
*15:25*	0.0223	0.0426	1.0000	0.0200	0.0400	1.0000	0.0250	0.0500	1.0000	0.0300	0.0600	1.0000	0.0100	0.0200	1.0000	0.0286	0.0429	1.0000
*15:31*	0.0021	0.0043	1.0000	0.0100	0.0200	1.0000												
*15:32*	0.0043	0.0085	1.0000	0.0100	0.0200	1.0000				0.0100	0.0200	1.0000						
*15:35*	0.0032	0.0064	1.0000	0.0050	0.0100	1.0000	0.0050	0.0100	1.0000	0.0050	0.0100	1.0000						
*18:01*	0.0383	0.0766	1.0000	0.0500	0.1000	1.0000	0.0150	0.0300	1.0000	0.0300	0.0600	1.0000	0.0600	0.1200	1.0000	0.0357	0.0714	1.0000
*18:02*	0.0160	0.0319	1.0000	0.0200	0.0400	1.0000	0.0100	0.0200	1.0000	0.0400	0.0800	1.0000				0.0071	0.0143	1.0000
*18:12*	0.0011	0.0021	1.0000				0.0050	0.0100	1.0000									
*18:14*	0.0011	0.0021	1.0000	0.0050	0.0100	1.0000												
*27:03*	0.0011	0.0021	1.0000				0.0050	0.0100	1.0000									
*27:04*	0.0213	0.0404	1.0000	0.0200	0.0400	1.0000	0.0400	0.0700	1.0000	0.0150	0.0300	1.0000	0.0100	0.0200	1.0000	0.0214	0.0429	1.0000
*27:06*	0.0138	0.0255	1.0000	0.0050	0.0100	1.0000	0.0200	0.0300	1.0000	0.0350	0.0700	1.0000	0.0050	0.0100	1.0000			
*27:07*	0.0011	0.0021	1.0000	0.0050	0.0100	1.0000												
*35:01*	0.0032	0.0064	1.0000	0.0050	0.0100	1.0000				0.0050	0.0100	1.0000				0.0071	0.0143	1.0000
*35:02*	0.0021	0.0043	1.0000										0.0050	0.0100	1.0000	0.0071	0.0143	1.0000
*35:03*	0.0074	0.0149	1.0000	0.0200	0.0400	1.0000				0.0050	0.0100	1.0000	0.0050	0.0100	1.0000	0.0071	0.0143	1.0000
*35:05*	0.0191	0.0383	1.0000	0.0300	0.0600	1.0000	0.0200	0.0400	1.0000	0.0200	0.0400	1.0000	0.0100	0.0200	1.0000	0.0143	0.0286	1.0000
*35:06*	0.0011	0.0021	1.0000	0.0050	0.0100	1.0000												
*35:11*	0.0011	0.0021	1.0000	0.0050	0.0100	1.0000												
*35:32*	0.0021	0.0043	1.0000	0.0100	0.0200	1.0000												
*35:68*	0.0011	0.0021	1.0000	0.0050	0.0100	1.0000												
*37:01*	0.0085	0.0170	1.0000	0.0150	0.0300	1.0000				0.0050	0.0100	1.0000	0.0050	0.0100	1.0000	0.0214	0.0429	1.0000
*38:02*	0.0426	0.0830	1.0000	0.0400	0.0800	1.0000	0.0550	0.1100	1.0000	0.0300	0.0600	1.0000	0.0500	0.0900	1.0000	0.0357	0.0714	1.0000
*39:01*	0.0064	0.0128	0.5000	0.0050	0.0100	1.0000	0.0050	0.0100	1.0000	0.0050	0.0100	1.0000	0.0050	0.0100	1.0000	0.0143	0.0286	1.0000
*39:06*	0.0011	0.0021	1.0000	0.0050	0.0100	1.0000												
*39:09*	0.0160	0.0319	1.0000				0.0150	0.0300	1.0000	0.0300	0.0600	1.0000	0.0250	0.0500	1.0000	0.0071	0.0143	1.0000
*39:15*	0.0053	0.0106	1.0000	0.0150	0.0300	1.0000				0.0050	0.0100	1.0000				0.0071	0.0143	1.0000
*39:49*	0.0011	0.0021	1.0000										0.0050	0.0100	1.0000			
*40:01*	0.0660	0.1234	0.6870	0.0300	0.0600	1.0000	0.0750	0.1400	1.0000	0.0950	0.1700	1.0000	0.0650	0.1200	1.0000	0.0643	0.1286	1.0000
*40:02*	0.0074	0.0149	1.0000				0.0100	0.0200	1.0000	0.0150	0.0300	1.0000	0.0100	0.0200	1.0000			
*40:06*	0.0234	0.0468	1.0000	0.0450	0.0900	1.0000	0.0200	0.0400	1.0000	0.0150	0.0300	1.0000	0.0200	0.0400	1.0000	0.0143	0.0286	1.0000
*40:11*	0.0011	0.0021	1.0000	0.0050	0.0100	1.0000												
*41:01*	0.0011	0.0021	1.0000	0.0050	0.0100	1.0000												
*41:30*	0.0011	0.0021	1.0000	0.0050	0.0100	1.0000												
*44:02*	0.0021	0.0043	1.0000	0.0050	0.0100	1.0000	0.0050	0.0100	1.0000									
*44:03*	0.0447	0.0894	1.0000	0.0450	0.0900	1.0000	0.0350	0.0700	1.0000	0.0250	0.0500	1.0000	0.0550	0.1100	1.0000	0.0714	0.1429	1.0000
*46:01*	0.1404	0.2596	0.8170	0.0650	0.1300	1.0000	0.1850	0.3300	1.0000	0.1300	0.2300	1.0000	0.1900	0.3600	0.6830	0.1286	0.2429	1.0000
*46:02*	0.0021	0.0043	1.0000							0.0050	0.0100	1.0000				0.0071	0.0143	1.0000
*48:01*	0.0043	0.0085	1.0000	0.0050	0.0100	1.0000	0.0050	0.0100	1.0000	0.0050	0.0100	1.0000	0.0050	0.0100	1.0000			
*48:03*	0.0106	0.0213	1.0000				0.0050	0.0100	1.0000	0.0050	0.0100	1.0000	0.0250	0.0500	1.0000	0.0214	0.0429	1.0000
*51:01*	0.0426	0.0851	1.0000	0.0550	0.1100	1.0000	0.0500	0.1000	1.0000	0.0350	0.0700	1.0000	0.0350	0.0700	1.0000	0.0357	0.0714	1.0000
*51:02*	0.0138	0.0277	1.0000	0.0250	0.0500	1.0000	0.0100	0.0200	1.0000	0.0100	0.0200	1.0000	0.0100	0.0200	1.0000	0.0143	0.0286	1.0000
*52:01*	0.0330	0.0638	1.0000	0.0450	0.0800	1.0000	0.0350	0.0700	1.0000	0.0200	0.0400	1.0000	0.0250	0.0500	1.0000	0.0429	0.0857	1.0000
*53:01*	0.0011	0.0021	1.0000				0.0050	0.0100	1.0000									
*54:01*	0.0085	0.0170	1.0000				0.0100	0.0200	1.0000	0.0100	0.0200	1.0000	0.0150	0.0300	1.0000	0.0071	0.0143	1.0000
*55:01*	0.0011	0.0021	1.0000							0.0050	0.0100	1.0000						
*55:02*	0.0213	0.0426	1.0000	0.0100	0.0200	1.0000	0.0300	0.0600	1.0000	0.0200	0.0400	1.0000	0.0150	0.0300	1.0000	0.0357	0.0714	1.0000
*56:01*	0.0106	0.0191	1.0000	0.0100	0.0200	1.0000	0.0100	0.0200	1.0000	0.0150	0.0200	1.0000	0.0100	0.0200	1.0000	0.0071	0.0143	1.0000
*56:02*	0.0011	0.0021	1.0000													0.0071	0.0143	1.0000
*56:04*	0.0128	0.0255	1.0000	0.0050	0.0100	1.0000	0.0050	0.0100	1.0000	0.0200	0.0400	1.0000	0.0250	0.0500	1.0000	0.0071	0.0143	1.0000
*57:01*	0.0117	0.0234	1.0000	0.0100	0.0200	1.0000	0.0100	0.0200	1.0000	0.0100	0.0200	1.0000	0.0150	0.0300	1.0000	0.0143	0.0286	1.0000
*57:02*	0.0011	0.0021	1.0000													0.0071	0.0143	1.0000
*58:01*	0.0638	0.1213	1.0000	0.0600	0.1100	1.0000	0.0500	0.1000	1.0000	0.0800	0.1400	0.4980	0.0750	0.1500	1.0000	0.0500	0.1000	1.0000
*58:51*	0.0011	0.0021	1.0000	0.0050	0.0100	1.0000												

HLA-B, human leukocyte antigen-B; AF, allele frequency; CF, carrier frequency; HW, Hardy–Weinberg equilibrium; Significantly different p-value < 0.05.

**Table 3:** The frequency of *HLA* class I alleles in Thai population and five regions (n = 470).

HLA-C	Thailand (n = 470)			Southern (n = 100)			Northern (n = 100)			Northeastern (n = 100)			Central (n = 100)			Bangkok (n = 70)		
	AF	CF	HW (p-value)	AF	CF	HW (p-value)	AF	CF	HW (p-value)	AF	CF	HW (p-value)	AF	CF	HW (p-value)	AF	CF	HW (p-value)
*01:02*	0.1713	0.3043	0.5895	0.0750	0.1400	1.0000	0.2200	0.3800	0.7560	0.1700	0.3100	1.0000	0.2200	0.3900	1.0000	0.1714	0.3000	1.0000
*01:08*	0.0011	0.0021	1.0000	0.0050	0.0100	1.0000												
*02:02*	0.0011	0.0021	1.0000				0.0050	0.0100	1.0000									
*03:02*	0.0777	0.1468	1.0000	0.0750	0.1300	1.0000	0.0600	0.1200	1.0000	0.1000	0.1800	1.0000	0.0900	0.1800	1.0000	0.0571	0.1143	1.0000
*03:03*	0.0202	0.0362	0.4995	0.0200	0.0400	1.0000	0.0200	0.0400	1.0000	0.0250	0.0400	1.0000	0.0050	0.0100	1.0000	0.0357	0.0571	1.0000
*03:04*	0.0809	0.1404	0.0901	0.0450	0.0900	1.0000	0.1600	0.2500	0.3311	0.0800	0.1400	1.0000	0.0800	0.1500	1.0000	0.0214	0.0429	1.0000
*03:08*	0.0021	0.0043	1.0000				0.0050	0.0100	1.0000				0.0050	0.0100	1.0000			
*03:09*	0.0011	0.0021	1.0000				0.0050	0.0100	1.0000									
*03:17*	0.0021	0.0043	1.0000							0.0050	0.0100	1.0000				0.0071	0.0143	1.0000
*03:94*	0.0011	0.0021	1.0000										0.0050	0.0100	1.0000			
*04:01*	0.0468	0.0936	1.0000	0.0800	0.1600	1.0000	0.0300	0.0600	1.0000	0.0250	0.0500	1.0000	0.0300	0.0600	1.0000	0.0786	0.1571	1.0000
*04:03*	0.0426	0.0809	1.0000	0.0400	0.0800	1.0000	0.0450	0.0900	1.0000	0.0600	0.1100	1.0000	0.0200	0.0400	1.0000	0.0500	0.0857	1.0000
*04:06*	0.0117	0.0234	1.0000	0.0050	0.0100	1.0000	0.0050	0.0100	1.0000	0.0250	0.0500	1.0000	0.0200	0.0400	1.0000			
*05:01*	0.0032	0.0064	1.0000	0.0050	0.0100	1.0000	0.0050	0.0100	1.0000				0.0050	0.0100	1.0000			
*06:02*	0.0426	0.0851	1.0000	0.0600	0.1200	1.0000	0.0200	0.0400	1.0000	0.0400	0.0800	1.0000	0.0350	0.0700	1.0000	0.0643	0.1286	1.0000
*06:89*	0.0011	0.0021	1.0000				0.0050	0.0100	1.0000									
*07:01*	0.0638	0.1234	1.0000	0.0700	0.1200	1.0000	0.0400	0.0800	1.0000	0.0350	0.0700	1.0000	0.0850	0.1700	1.0000	0.1000	0.2000	1.0000
*07:02*	0.1191	0.2149	0.4752	0.0900	0.1600	1.0000	0.1000	0.1800	1.0000	0.1450	0.2600	1.0000	0.1400	0.2700	1.0000	0.1214	0.2000	0.6195
*07:04*	0.0500	0.0979	1.0000	0.0650	0.1300	1.0000	0.0300	0.0600	1.0000	0.0650	0.1200	1.0000	0.0350	0.0700	1.0000	0.0571	0.1143	1.0000
*07:27*	0.0117	0.0234	1.0000	0.0150	0.0300	1.0000	0.0200	0.0400	1.0000				0.0150	0.0300	1.0000	0.0071	0.0143	1.0000
*07:29*	0.0085	0.0170	1.0000				0.0050	0.0100	1.0000	0.0200	0.0400	1.0000	0.0100	0.0200	1.0000	0.0071	0.0143	1.0000
*08:01*	0.1032	0.1915	0.7714	0.1450	0.2700	1.0000	0.0850	0.1700	1.0000	0.0850	0.1500	1.0000	0.1150	0.2200	1.0000	0.0786	0.1286	0.4964
*08:02*	0.0021	0.0043	1.0000	0.0050	0.0100	1.0000										0.0071	0.0143	1.0000
*08:03*	0.0032	0.0064	1.0000				0.0050	0.0100	1.0000	0.0100	0.0200	1.0000						
*12:02*	0.0404	0.0787	1.0000	0.0550	0.1000	1.0000	0.0700	0.1400	1.0000	0.0150	0.0300	1.0000	0.0200	0.0400	1.0000	0.0429	0.0857	1.0000
*12:03*	0.0138	0.0277	1.0000	0.0200	0.0400	1.0000	0.0100	0.0200	1.0000	0.0200	0.0400	1.0000	0.0050	0.0100	1.0000	0.0143	0.0286	1.0000
*12:04*	0.0011	0.0021	1.0000							0.0050	0.0100	1.0000						
*14:02*	0.0287	0.0574	1.0000	0.0350	0.0700	1.0000	0.0300	0.0600	1.0000	0.0300	0.0600	1.0000	0.0250	0.0500	1.0000	0.0214	0.0429	1.0000
*15:02*	0.0340	0.0681	1.0000	0.0600	0.1200	1.0000	0.0150	0.0300	1.0000	0.0250	0.0500	1.0000	0.0300	0.0600	1.0000	0.0429	0.0857	1.0000
*15:05*	0.0043	0.0085	1.0000				0.0050	0.0100	1.0000	0.0150	0.0300	1.0000						
*16:02*	0.0064	0.0128	1.0000	0.0200	0.0400	1.0000							0.0050	0.0100	1.0000	0.0071	0.0143	1.0000
*17:01*	0.0021	0.0043	1.0000	0.0100	0.0200	1.0000												
*18:01*	0.0011	0.0021	1.0000													0.0071	0.0143	1.0000

HLA-C, human leukocyte antigen-C; AF, allele frequency; CF, carrier frequency; HW, Hardy–Weinberg equilibrium; Significantly different p-value < 0.05.

**Table 4:** The frequency of *HLA* class II alleles in Thai population and five regions (n = 470).

HLA-DRB1	Thailand (n = 470)			Southern (n = 100)			Northern (n = 100)			Northeastern (n = 100)			Central (n = 100)			Bangkok (n = 70)		
	AF	CF	HW (p-value)	AF	CF	HW (p-value)	AF	CF	HW (p-value)	AF	CF	HW (p-value)	AF	CF	HW (p-value)	AF	CF	HW (p-value)
*01:01*	0.0032	0.0064	1.0000	0.0050	0.0100	1.0000							0.0100	0.0200	1.0000			
*01:02*	0.0011	0.0021	1.0000													0.0071	0.0143	1.0000
*03:01*	0.0500	0.0915	0.3737	0.0500	0.0900	1.0000	0.0400	0.0800	1.0000	0.0650	0.1100	1.0000	0.0500	0.1000	1.0000	0.0429	0.0714	1.0000
*03:20*	0.0032	0.0064	1.0000				0.0050	0.0100	1.0000							0.0143	0.0286	1.0000
*04:01*	0.0032	0.0064	1.0000	0.0050	0.0100	1.0000	0.0050	0.0100	1.0000							0.0071	0.0143	1.0000
*04:02*	0.0011	0.0021	1.0000	0.0050	0.0100	1.0000												
*04:03*	0.0234	0.0447	1.0000	0.0300	0.0600	1.0000	0.0150	0.0300	1.0000	0.0050	0.0100	1.0000	0.0250	0.0500	1.0000	0.0500	0.0857	1.0000
*04:04*	0.0032	0.0064	1.0000	0.0150	0.0300	1.0000												
*04:05*	0.0489	0.0957	1.0000	0.0150	0.0300	1.0000	0.0500	0.1000	1.0000	0.0850	0.1600	1.0000	0.0650	0.1300	1.0000	0.0214	0.0429	1.0000
*04:06*	0.0032	0.0064	1.0000	0.0050	0.0100	1.0000							0.0050	0.0100	1.0000	0.0071	0.0143	1.0000
*05:01*	0.0011	0.0021	1.0000	0.0050	0.0100	1.0000												
*07:01*	0.0894	0.1766	0.3737	0.0850	0.1600	1.0000	0.0600	0.1200	1.0000	0.0750	0.1500	1.0000	0.1100	0.2200	1.0000	0.1286	0.2571	1.0000
*08:01*	0.0011	0.0021	1.0000	0.0050	0.0100	1.0000												
*08:02*	0.0021	0.0043	1.0000	0.0050	0.0100	1.0000										0.0071	0.0143	1.0000
*08:03*	0.0160	0.0298	1.0000	0.0300	0.0600	1.0000	0.0050	0.0100	1.0000	0.0150	0.0300	1.0000	0.0050	0.0100	1.0000	0.0286	0.0429	1.0000
*08:12*	0.0021	0.0043	1.0000	0.0050	0.0100	1.0000							0.0050	0.0100	1.0000			
*08:19*	0.0011	0.0021	1.0000										0.0050	0.0100	1.0000			
*09:01*	0.0989	0.1872	1.0000	0.0800	0.1600	1.0000	0.1350	0.2600	1.0000	0.1100	0.2200	1.0000	0.0650	0.1200	1.0000	0.1071	0.1714	0.6195
*10:01*	0.0213	0.0426	1.0000	0.0300	0.0600	1.0000	0.0200	0.0400	1.0000	0.0200	0.0400	1.0000	0.0150	0.0300	1.0000	0.0214	0.0429	1.0000
*11:01*	0.0160	0.0319	1.0000	0.0200	0.0400	1.0000	0.0100	0.0200	1.0000	0.0150	0.0300	1.0000	0.0200	0.0400	1.0000	0.0143	0.0286	1.0000
*11:02*	0.0011	0.0021	1.0000	0.0050	0.0100	1.0000												
*11:04*	0.0021	0.0043	1.0000	0.0100	0.0200	1.0000												
*11:05*	0.0021	0.0043	1.0000	0.0050	0.0100	1.0000							0.0050	0.0100	1.0000			
*11:06*	0.0138	0.0277	1.0000	0.0100	0.0200	1.0000				0.0200	0.0400	1.0000	0.0250	0.0500	1.0000	0.0143	0.0286	1.0000
*12:01*	0.0074	0.0149	1.0000	0.0050	0.0100	1.0000	0.0100	0.0200	1.0000				0.0150	0.0300	1.0000	0.0071	0.0143	1.0000
*12:02*	0.1532	0.2851	0.8253	0.1300	0.2500	1.0000	0.1350	0.2600	1.0000	0.1600	0.3100	0.6212	0.1650	0.2900	1.0000	0.1857	0.3286	1.0000
*12:07*	0.0032	0.0064	1.0000				0.0050	0.0100	1.0000	0.0050	0.0100	1.0000				0.0071	0.0143	1.0000
*12:12*	0.0011	0.0021	1.0000				0.0050	0.0100	1.0000									
*12:16*	0.0021	0.0043	1.0000	0.0050	0.0100	1.0000										0.0071	0.0143	1.0000
*13:01*	0.0096	0.0191	1.0000	0.0200	0.0400	1.0000				0.0050	0.0100	1.0000	0.0050	0.0100	1.0000	0.0214	0.0429	1.0000
*13:02*	0.0138	0.0277	1.0000	0.0300	0.0600	1.0000				0.0150	0.0300	1.0000	0.0100	0.0200	1.0000	0.0143	0.0286	1.0000
*13:12*	0.0074	0.0149	1.0000				0.0050	0.0100	1.0000	0.0150	0.0300	1.0000				0.0214	0.0429	1.0000
*14:01*	0.0596	0.1085	1.0000	0.0400	0.0700	1.0000	0.1150	0.2000	0.6195	0.0350	0.0700	1.0000	0.0650	0.1200	1.0000	0.0357	0.0714	1.0000
*14:03*	0.0011	0.0021	1.0000	0.0050	0.0100	1.0000												
*14:04*	0.0234	0.0447	1.0000	0.0250	0.0500	1.0000	0.0150	0.0300	1.0000	0.0300	0.0500	1.0000	0.0300	0.0600	1.0000	0.0143	0.0286	1.0000
*14:05*	0.0074	0.0149	1.0000				0.0150	0.0300	1.0000	0.0050	0.0100	1.0000				0.0214	0.0429	1.0000
*14:07*	0.0011	0.0021	1.0000	0.0050	0.0100	1.0000												
*14:22*	0.0021	0.0043	1.0000										0.0050	0.0100	1.0000	0.0071	0.0143	1.0000
*15:01*	0.0809	0.1532	1.0000	0.1050	0.2000	1.0000	0.0750	0.1500	1.0000	0.0650	0.1200	1.0000	0.0950	0.1800	1.0000	0.0571	0.1000	1.0000
*15:02*	0.1447	0.2638	0.8292	0.1600	0.3100	0.6212	0.1650	0.2900	1.0000	0.1700	0.3100	1.0000	0.1250	0.2200	1.0000	0.0857	0.1571	1.0000
*15:03*	0.0011	0.0021	1.0000													0.0071	0.0143	1.0000
*15:06*	0.0011	0.0021	1.0000	0.0050	0.0100	1.0000												
*15:24*	0.0011	0.0021	1.0000							0.0050	0.0100	1.0000						
*16:01*	0.0011	0.0021	1.0000				0.0050	0.0100	1.0000									
*16:02*	0.0596	0.1106	0.6865	0.0350	0.0700	1.0000	0.1000	0.1600	1.0000	0.0700	0.1400	1.0000	0.0650	0.1300	1.0000	0.0143	0.0286	1.0000
*16:05*	0.0021	0.0043	1.0000							0.0100	0.0200	1.0000						
*16:10*	0.0011	0.0021	1.0000													0.0071	0.0143	1.0000
*16:12*	0.0064	0.0128	1.0000	0.0050	0.0100	1.0000	0.0050	0.0100	1.0000				0.0100	0.0200	1.0000	0.0143	0.0286	1.0000

HLA-DRB1, human leukocyte antigen-DRB1; AF, allele frequency; CF, carrier frequency; HW, Hardy–Weinberg equilibrium; Significantly different p-value < 0.05.

**Table 5:** The frequency of *HLA* class II alleles in Thai population and five regions (n = 470).

HLA-DQA1	Thailand (n = 470)			Southern (n = 100)			Northern (n = 100)			Northeastern (n = 100)			Central (n = 100)			Bangkok (n = 70)		
	AF	CF	HW (p-value)	AF	CF	HW (p-value)	AF	CF	HW (p-value)	AF	CF	HW (p-value)	AF	CF	HW (p-value)	AF	CF	HW (p-value)
*01:01*	0.2489	0.4170	0.3105	0.2350	0.4000	0.7740	0.3250	0.5000	0.5280	0.2450	0.4300	1.0000	0.2400	0.4200	1.0000	0.1786	0.3000	0.6810
*01:02*	0.2223	0.3894	0.7692	0.2150	0.3900	1.0000	0.2150	0.3700	0.7560	0.2600	0.4300	0.7940	0.2200	0.3900	1.0000	0.1929	0.3571	1.0000
*01:03*	0.0383	0.0723	1.0000	0.0850	0.1600	1.0000	0.0150	0.0300	1.0000	0.0150	0.0300	1.0000	0.0350	0.0700	1.0000	0.0429	0.0714	1.0000
*01:06*	0.0011	0.0021	1.0000													0.0071	0.0143	1.0000
*02:01*	0.0872	0.1723	0.3740	0.0800	0.1500	1.0000	0.0600	0.1200	1.0000	0.0700	0.1400	1.0000	0.1100	0.2200	1.0000	0.1286	0.2571	1.0000
*03:01*	0.0457	0.0851	0.6240	0.0650	0.1200	1.0000	0.0350	0.0700	1.0000	0.0300	0.0600	1.0000	0.0400	0.0700	1.0000	0.0643	0.1143	1.0000
*03:02*	0.1330	0.2660	0.0080	0.0850	0.1700	1.0000	0.1700	0.3400	0.2460	0.1700	0.3400	0.2460	0.1250	0.2500	0.4980	0.1071	0.2143	1.0000
*04:01*	0.0021	0.0043	1.0000				0.0050	0.0100	1.0000							0.0071	0.0143	1.0000
*05:01*	0.0543	0.1043	1.0000	0.0600	0.1100	1.0000	0.0450	0.0900	1.0000	0.0600	0.1100	1.0000	0.0500	0.1000	1.0000	0.0571	0.1143	1.0000
*05:03*	0.0117	0.0234	1.0000	0.0050	0.0100	1.0000	0.0050	0.0100	1.0000	0.0150	0.0300	1.0000	0.0150	0.0300	1.0000	0.0214	0.0429	1.0000
*05:05*	0.0340	0.0660	1.0000	0.0350	0.0600	1.0000	0.0150	0.0300	1.0000	0.0400	0.0800	1.0000	0.0450	0.0900	1.0000	0.0357	0.0714	1.0000
*05:08*	0.0011	0.0021	1.0000				0.0050	0.0100	1.0000									
*06:01*	0.1202	0.2277	0.7800	0.1350	0.2600	1.0000	0.1050	0.2100	1.0000	0.0950	0.1800	1.0000	0.1200	0.2200	1.0000	0.1571	0.2857	1.0000

HLA-DQA1, human leukocyte antigen-DQA1; AF, allele frequency; CF, carrier frequency; HW, Hardy–Weinberg equilibrium; Significantly different p-value < 0.05.

**Table 6:** The frequency of *HLA* class II alleles in Thai population and five regions (n = 470).

HLA-DQB1	Thailand (n = 470)			Southern (n = 100)			Northern (n =100)			Northeastern (n = 100)			Central (n = 100)			Bangkok (n = 70)		
	AF	CF	HW (p-value)	AF	CF	HW (p-value)	AF	CF	HW (p-value)	AF	CF	HW (p-value)	AF	CF	HW (p-value)	AF	CF	HW (p-value)
*02:01*	0.0543	0.1000	0.3740	0.0550	0.1000	1.0000	0.0450	0.0900	1.0000	0.0650	0.1100	0.4980	0.0500	0.1000	1.0000	0.0571	0.1000	1.0000
*02:02*	0.0723	0.1447	0.5000	0.0700	0.1400	1.0000	0.0500	0.1000	1.0000	0.0600	0.1200	1.0000	0.0850	0.1700	1.0000	0.1071	0.2143	1.0000
*03:01*	0.1723	0.3213	0.6850	0.1850	0.3300	1.0000	0.1400	0.2700	1.0000	0.1500	0.2900	1.0000	0.1800	0.3400	1.0000	0.2214	0.4000	1.0000
*03:02*	0.0426	0.0787	0.6240	0.0600	0.1100	1.0000	0.0300	0.0600	1.0000	0.0200	0.0400	1.0000	0.0400	0.0700	1.0000	0.0714	0.1286	1.0000
*03:03*	0.1128	0.2149	0.7620	0.0850	0.1700	1.0000	0.1350	0.2600	1.0000	0.1250	0.2500	1.0000	0.0950	0.1800	1.0000	0.1286	0.2143	0.6200
*03:38*	0.0011	0.0021	1.0000	0.0050	0.0100	1.0000												
*04:01*	0.0383	0.0745	1.0000	0.0150	0.0300	1.0000	0.0400	0.0800	1.0000	0.0600	0.1100	1.0000	0.0550	0.1100	1.0000	0.0143	0.0286	1.0000
*04:02*	0.0032	0.0064	1.0000				0.0050	0.0100	1.0000	0.0050	0.0100	1.0000				0.0071	0.0143	1.0000
*05:01*	0.1404	0.2574	0.8210	0.1350	0.2600	1.0000	0.1700	0.2900	0.7210	0.1600	0.3000	1.0000	0.1350	0.2500	1.0000	0.0857	0.1571	1.0000
*05:02*	0.2128	0.3872	0.7440	0.1600	0.2800	1.0000	0.2850	0.4900	1.0000	0.2400	0.4500	0.4980	0.2000	0.3900	0.3690	0.1643	0.3000	1.0000
*05:03*	0.0404	0.0787	1.0000	0.0450	0.0900	1.0000	0.0400	0.0800	1.0000	0.0350	0.0600	1.0000	0.0500	0.1000	1.0000	0.0286	0.0571	1.0000
*05:66*	0.0011	0.0021	1.0000	0.0050	0.0100	1.0000												
*06:01*	0.0713	0.1340	0.6870	0.1150	0.2100	1.0000	0.0400	0.0800	1.0000	0.0550	0.1100	1.0000	0.0850	0.1600	1.0000	0.0571	0.1000	1.0000
*06:02*	0.0149	0.0298	1.0000	0.0150	0.0300	1.0000	0.0150	0.0300	1.0000	0.0100	0.0200	1.0000	0.0100	0.0200	1.0000	0.0286	0.0571	1.0000
*06:03*	0.0074	0.0149	1.0000	0.0200	0.0400	1.0000							0.0050	0.0100	1.0000	0.0143	0.0286	1.0000
*06:04*	0.0053	0.0106	1.0000	0.0100	0.0200	1.0000				0.0050	0.0100	1.0000	0.0050	0.0100	1.0000	0.0071	0.0143	1.0000
*06:05*	0.0043	0.0085	1.0000	0.0050	0.0100	1.0000				0.0050	0.0100	1.0000	0.0050	0.0100	1.0000	0.0071	0.0143	1.0000
*06:09*	0.0043	0.0085	1.0000	0.0150	0.0300	1.0000				0.0050	0.0100	1.0000						
*06:10*	0.0011	0.0021	1.0000				0.0050	0.0100	1.0000									

HLA-DQB1, human leukocyte antigen-DQB1; AF, allele frequency; CF, carrier frequency; HW, Hardy–Weinberg equilibrium; Significantly different p-value < 0.05.

**Table 7:** Distribution of pharmacogenetics markers in five regions and Thai population.

Drug	Pharmacogenetics markers	ADR type	Allele frequency (%)						Comparing all five populations (*p*-value)	Reference	
			Thai population (n = 470)	Southern (n = 100)	Northern (n = 100)	Northeastern (n = 100)	Central (n = 100)	Bangkok (n = 70)			
Carbamazepine	HLA-B*15:02	SJS/TEN	7.66	9.00	6.50	6.50	7.50	9.29	0.9052	(Nahoko and Yoshiro, 2013; Sukasem et al., 2014; Su et al., 2016)	
	HLA-A*31:01	CADRs, SJS/TEN, DRESS, MPE	0.85	2.00	0.50	0.00	0.5	1.43	0.8050	(Nahoko and Yoshiro, 2013; Sukasem et al., 2014; Su et al., 2016)	
	HLA-B*15:11	SJS/TEN	0.21	0.50	0.50	0.00	0.00	0.00	1.0000	(Sukasem et al., 2014; Su et al., 2016)	
	HLA-A*24:02	SJS/TEN	11.49	14.50	10.00	11.50	10.00	11.43	0.8546	(Shi et al., 2017)	
	HLA-C*08:01	SJS/TEN	10.32	14.50	8.50	8.50	11.50	7.86	0.4764	(Shi et al., 2017)	
	HLA-DRB1*12:02	SJS/TEN	15.32	13.00	13.50	16.00	16.50	18.57	0.8070	(Shi et al., 2017)	
Oxcarbazepine	HLA-B*15:02	MPE, SJS	7.66	9.00	6.50	6.50	7.50	9.29	0.9052	(Su et al., 2016)	
	HLA-B*13:02	MPE	2.13	3.50	1.50	1.50	1.50	2.86	0.8830	(Lauren et al., 2014)	
	HLA-B*38:02	MPE	4.26	4.00	5.50	3.00	5.00	3.57	0.3380	(Lv et al., 2013)	
Phenytoin	HLA-B*15:02	SJS/TEN	7.66	9.00	6.50	6.50	7.50	9.29	0.9052	(Lauren et al., 2014; Su et al., 2016)	
	HLA-A*24:02	SJS/TEN	11.49	14.50	10.00	11.50	10.00	11.43	0.8546	(Shi et al., 2017)	
	HLA-B*13:01	SCARs	5.96	3.50	8.00	6.00	6.50	5.71	0.7450	(Yampayon et al., 2017)	
	HLA-B*56:02	DRESS	0.11	0.00	0.00	0.00	0.00	0.71	1.0000	(Yampayon et al., 2017)	
	HLA-B*15:13	SJS/TEN, DRESS	0.96	2.50	0.00	0.50	1.00	0.71	0.1330	(Hung et al., 2010; Jaruthamsophon et al., 2016; Chang et al., 2017)	
	HLA-C*08:01	SJS/TEN	10.32	14.50	8.50	8.50	11.50	7.86	0.4764	(Hung et al., 2010; White et al., 2014)	
	HLA-DRB1*16:02	**SJS/TEN**	**5.96**	**3.50**	**10.00**	**7.00**	**6.50**	**1.43**	**0.0470**	(Hung et al., 2010; White et al., 2014)	
Lamotrigine	HLA-A*24:02	SJS/TEN, MPE	11.49	14.50	10.00	11.50	10.00	11.43	0.8546	(Moon et al., 2015; Shi et al., 2017)	
	HLA-A*31:01	SCARs	0.85	2.00	0.50	0.00	0.5	1.43	0.8050	(Kim et al., 2017)	
	HLA-A*68:01	SCARs	0.96	2.00	0.00	1.50	0.00	1.43	0.5730	(Kazeem et al., 2009)	
Phenobarbital	HLA-B*51:01	SJS/TEN	4.26	5.50	5.00	3.50	3.50	3.57	0.9640	(White et al., 2014)	
	HLA-A*01:01	SCARs, MPE	2.23	4.50	0.50	0.50	2.50	3.57	0.3250	(Manuyakorn et al., 2016)	
	HLA-B*13:01	SCARs	5.96	3.50	8.00	6.00	6.50	5.71	0.7450	(Manuyakorn et al., 2016)	
Allopurinol	HLA-B*58:01	CADRs, SCARs, MPE, SJS/TEN, DRESS	6.38	6.00	5.00	8.00	7.50	5.00	0.8528	(Cristallo et al., 2011; Sukasem et al., 2014; Su et al., 2016; Sukasem et al., 2016)	
	HLA-C*03:02	SJS/TEN	7.77	7.50	6.00	10.00	9.00	5.71	0.7394	(Cristallo et al., 2011; Li et al., 2017)	
	HLA-A*33:03	SJS/TEN	11.17	10.50	8.50	10.00	14.50	12.86	0.6808	(Cristallo et al., 2011; Li et al., 2017)	
	HLA-C*08:01	SJS/TEN	10.32	14.50	8.50	8.50	11.50	7.86	0.4764	(Cristallo et al., 2011)	
	HLA-DRB1*13:02	SJS/TEN	1.38	3.00	0.00	1.50	1.00	1.43	0.5520	(Cristallo et al., 2011)	
	HLA-DRB1*15:02	SJS/TEN	14.47	16.00	16.50	17.00	12.50	8.57	0.3745	(Cristallo et al., 2011)	
Abacavir	HLA-B*57:01	AHS	1.17	1.00	1.00	1.00	1.50	1.43	1.0000		(Sukasem et al., 2014; Dao et al., 2015; Su et al., 2016)
Nevirapine	HLA-B*35:05	SJS/TEN, DRESS	1.91	3.00	2.00	2.00	1.00	1.43	0.9400		(Chantarangsu et al., 2009; Sukasem et al., 2014; Lauren et al., 2014)
Co-trimoxazole	HLA-B*38:01	SJS/TEN	0.00	0.00	0.00	0.00	0.00	0.00	N/A		(Lonjou et al., 2008; White et al., 2014)
	HLA-B*38:02	SJS/TEN	4.26	4.00	5.50	3.00	5.00	3.57	0.9230		(Lonjou et al., 2008; White et al., 2014)
	HLA-B*15:02	SJS/TEN	7.66	9.00	6.50	6.50	7.50	9.29	0.9052		(Kongpan et al., 2015)
	HLA-C*06:02	SJS/TEN	4.26	6.00	2.00	4.00	3.50	6.43	0.5100		(Kongpan et al., 2015)
	HLA-C*08:01	SJS/TEN	10.32	14.50	8.50	8.50	11.50	7.86	0.4764		(Kongpan et al., 2015)
Dapsone	HLA-B*13:01	SCAR DRESS	5.96	3.50	8.00	6.00	6.50	5.71	0.7450		(Zhang et al., 2013; White et al., 2014; Tempark et al., 2017)
Salazosulfa- Pyridine	HLA-B*13:01	DRESS	5.96	3.50	8.00	6.00	6.50	5.71	0.7450		(Yang et al., 2014)
Methazolamide	HLA-B*59:01	SJS/TEN	0.00	0.00	0.00	0.00	0.00	0.00	N/A		(White et al., 2014; Yang et al., 2016)
Amoxicillin– Clavulanate	HLA-A*30:02	DILI	0.00	0.00	0.00	0.00	0.00	0.00	N/A		(Stephens et al., 2013)
	HLA-DRB1*15:01	DILI	8.09	10.50	7.50	6.50	9.50	5.71	0.6862		(Lucena et al., 2011; Stephens et al., 2013)
	HLA-DQB1*06:02	DILI	1.49	1.50	1.50	1.00	1.00	2.86	0.9400		(Lucena et al., 2011; Stephens et al., 2013)
Ticlopidine	HLA-A*33:03	DILI	11.17	10.50	8.50	10.00	14.50	12.86	0.6808		(Hirata et al., 2008)
Flucloxacillin	HLA-B*57:01	DILI	1.17	1.00	1.00	1.00	1.50	1.43	1.0000		(Daly et al., 2009)
Lapatinib	HLA-DQA1*02:01	DILI	8.72	8.00	6.00	7.00	11.00	12.86	0.3900		(Spraggs et al., 2011)

ADR, adverse drug reactions; HLA-A, human leukocyte antigen-A; HLA-B, human leukocyte antigen-B; HLA-C, human leukocyte antigen-C; HLA-DRB1, human leukocyte antigen-DRB1; HLA-DQA1, human leukocyte antigen-DQA1; HLA-DQB1, human leukocyte antigen-DQB1; AHS, abacavir hypersensitivity; CADRs, cutaneous adverse drug reactions; DRESS, drug reactions with eosinophilia and systemic symptoms; DILI, drug-induced liver injury; MPE, maculopapular exanthema; SCARs, severe cutaneous adverse reactions; SJS, Stevens–Johnson syndrome; TEN, toxic epidermal necrolysis; N/A, not available; Significant difference p-value < 0.05 by Pearson’s chi-square test.

Bolded text: Data analysis result was presented statistical significance (p-value < 0.05).

The frequency of *HLA* class II alleles including *HLA-DRB1*, *HLA-DQA1*, and *HLA-DQB1* alleles were presented in **Tables 4**–**6**. The frequency distributions of *HLA-DRB1* alleles were HLA-DRB1*12:02 (15.32%), -DRB1*15:02 (14.47%), -DRB1*09:01 (9.89%), -DRB1*07:01 (8.94%), -DRB1*15:01 (8.09%), -DRB1*14:01 (5.96%), -DRB1*16:02 (5.96%), and -DRB1*03:01 (5.00%). Particularly, HLA-DRB1*15:02 was the highest allele frequency presented in the Southern, Northern, and Northeastern groups, while Central and Bangkok regions share the top allele frequency of HLA-DRB1*12:02. Moreover, frequencies of *HLA-DQA1* alleles were HLA-DQA1*01:01 (24.89%), -DQA1*01:02 (22.23%), -DQA1*03:02 (13.30%), -DQA1*06:01 (12.02%), -DQA1*02:01 (8.72%), and -DQA1*05:01 (5.43%) (**Table 5**). HLA-DQA1*01:01 allele was the highest allele in the Southern, Northern, and Central groups. On the contrary, HLA-DQA1*01:02 allele was found more commonly in both Northeastern and Bangkok regions. The frequency distribution of *HLA-DQB1* alleles includes HLA-DQB1*05:02 (21.28%), -DQB1*03:01 (17.23%), -DQB1*05:01 (14.04%), -DQB1*03:03 (11.28%), -DQB1*02:02 (7.23%), -DQB1*06:01 (7.13%), and -DQB1*02:01 (5.43%), as shown in **Table 6**. HLA-DQB1*03:01 was the main allele in both Southern and Bangkok regions meanwhile HLA-DQB1*05:02 was found more common in Northern, Northeastern, and Central. For the statistical analysis, the allele frequency of *HLA-A*, *-B*, *-C*, *-DRB1*, *-DQA1*, and *-DQB1* were tested from Hardy–Weinberg equilibrium (*p*-value < 0.05). There was no significant differentiation in each *HLA* class I and class II alleles except only HLA-DQA1*03:02 allele in Thai population.

### Distribution of Pharmacogenetics Markers

**Figure 1:**
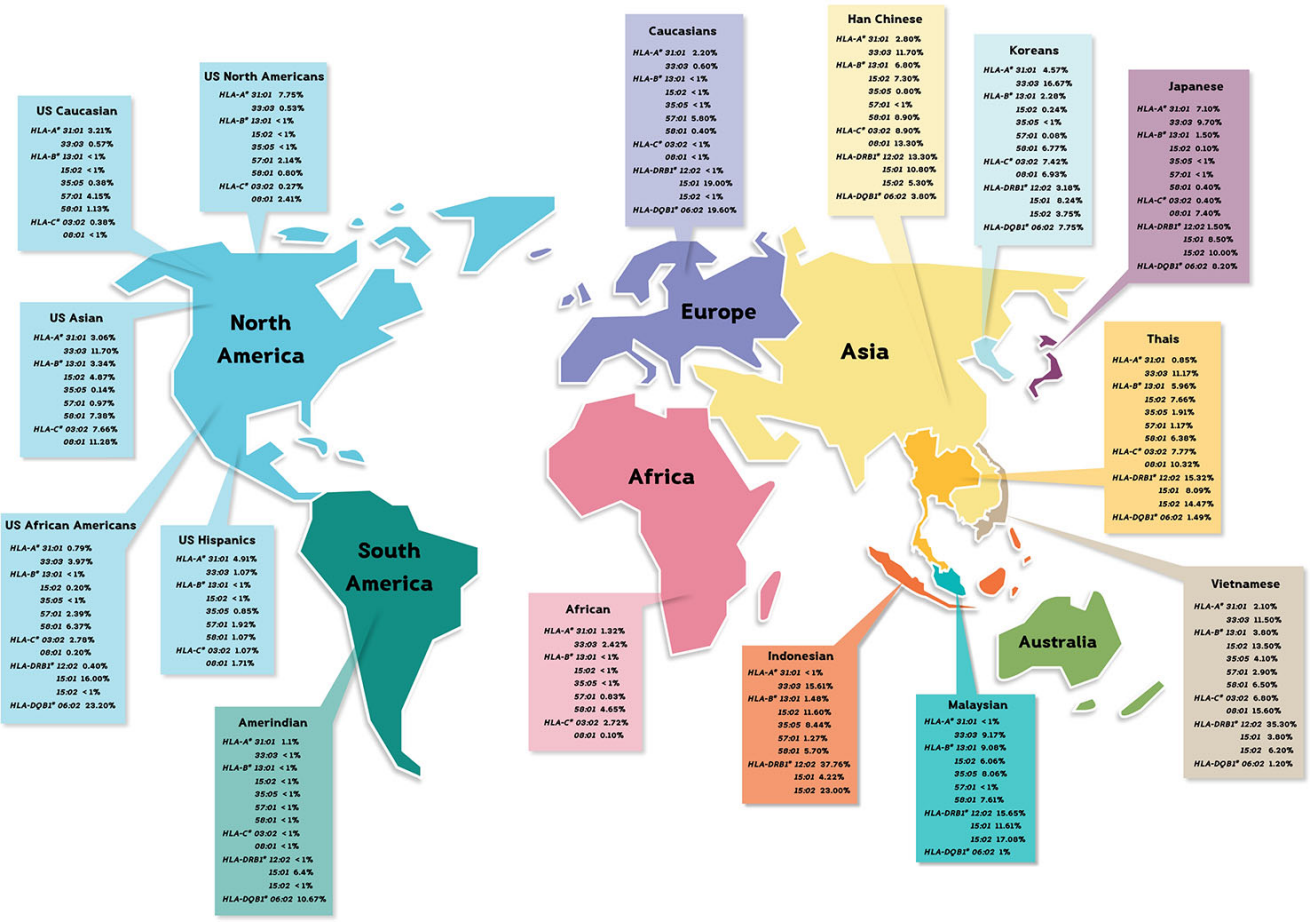
The distribution of pharmacogenetics markers in many populations.

The allele frequencies across Thailand five regions of HLA-B*15:02 (IMGT/HLA ID: HLA00165) which induces the risk of having SJS/TEN upon taking aromatic antiepileptic drug [CBZ, oxcarbazepine (OXC), phenytoin (PHT), and lamotrigine (LTG)], had no significant differences (*p*-value > 0.05) (**Table 7**). The frequency of HLA-B*15:02 allele was much lower in other populations, namely, African Americans (Cao et al., 2001), Caucasians (Cao et al., 2001), Hispanics (Cao et al., 2001), and North Americans (Cao et al., 2001) (**Table 8A** and **Figure 1**). Additionally, *HLA-B∗15:02* allele belongs to the HLA-B75 family, which consists of *HLA-B∗15:11* (IMGT/HLA ID: HLA00174) (0.50%) and *HLA-B∗15:21* (IMGT/HLA ID: HLA00184) (0.50%) in Southern group, whereas *HLA-B∗15:08* (IMGT/HLA ID: HLA00171) was not found in this population. In terms of HLA-A*31:01 (IMGT/HLA ID: HLA00097) allele which associates with CBZ-induced CADRs, neither the frequencies within Thailand nor among different populations were significantly different (**Tables 7**, **8A**).

**Table 8A:** Comparison of pharmacogenetics markers in Thai and other populations.

Drug	Pharmacogenetics markers	ADR type	Allele frequency (%)						Reference
			Thai population (n = 470)	African Americans (n = 252) (Cao et al., 2001)	Caucasians (n = 265) (Cao et al., 2001)	Hispanics (n = 234) (Cao et al., 2001)	North American (n = 187) (Cao et al., 2001)	Asians (n = 358) (Cao et al., 2001)	
Carbamazepine	HLA-B*15:02	SJS/TEN	7.66	0.20	0.00	0.00	0.00	4.87	(Nahoko and Yoshiro, 2013; Sukasem et al., 2014; Su et al., 2016)	
	HLA-A*31:01	CADRs, SJS/TEN, DRESS, MPE	0.85	0.79	3.21	4.91	7.75	3.06	(Nahoko and Yoshiro, 2013; Sukasem et al., 2014; Su et al., 2016)	
	HLA-B*15:11	SJS/TEN	0.21	0.00	0.00	0.00	0.00	0.28	(Sukasem et al., 2014; Su et al., 2016)	
	HLA-A*24:02	SJS/TEN	11.49	2.78	6.60	12.18	22.73	18.94	(Shi et al., 2017)	
	HLA-C*08:01	SJS/TEN	10.32	0.20	0.00	1.71	2.41	11.28	(Shi et al., 2017)	
Oxcarbazepine	HLA-B*15:02	MPE, SJS	7.66	0.20	0.00	0.00	0.00	4.87	(Su et al., 2016)	
	HLA-B*13:02	MPE	2.13	1.20	1.32	1.50	1.87	1.95	(Lauren et al., 2014)	
	HLA-B*38:02	MPE	4.26	0.00	0.19	0.00	0.00	6.55	(Lv et al., 2013)	
Phenytoin	HLA-B*15:02	SJS/TEN	7.66	0.20	0.00	0.00	0.00	4.87	(Lauren et al., 2014; Su et al., 2016)	
	HLA-A*24:02	SJS/TEN	11.49	2.78	6.60	12.18	22.73	18.94	(Shi et al., 2017)	
	HLA-B*13:01	SCARs	5.96	0.00	0.00	0.00	0.00	3.34	(Yampayon et al., 2017)	
	HLA-B*56:02	DRESS	0.11	0.00	0.00	0.00	0.00	0.28	(Yampayon et al., 2017)	
	HLA-B*15:13	SJS/TEN, DRESS	0.96	0.00	0.00	0.00	0.00	0.28	(Hung et al., 2010; Jaruthamsophon et al., 2016; Chang et al., 2017)	
	HLA-C*08:01	SJS/TEN	10.32	0.20	0.00	1.71	2.41	11.28	(Hung et al., 2010; White et al., 2014)	
Lamotrigine	HLA-A*24:02	SJS/TEN,MPE	11.49	2.78	6.60	12.18	22.73	18.94	(Moon et al., 2015; Shi et al., 2017)	
	HLA-A*31:01	SCARs	0.85	0.79	3.21	4.91	7.75	3.06	(Kim et al., 2017)	
	HLA-A*68:01	SCARs	0.96	2.58	3.02	2.56	5.62	0.28	(Kazeem et al., 2009)	
Phenobarbital	HLA-B*51:01	SJS/TEN	4.26	1.20	5.66	6.20	11.23	6.69	(White et al., 2014)	
	HLA-A*01:01	SCARs, MPE	2.23	5.56	15.09	5.98	7.49	1.53	(Manuyakorn et al., 2016)	
	HLA-B*13:01	DRESS	5.96	0.00	0.00	0.00	0.00	3.34	(Manuyakorn et al., 2016)	
Allopurinol	HLA-B*58:01	CADRs, SCARs, MPE, SJS/TEN, DRESS	6.38	6.37	1.13	1.07	0.80	7.38	(Cristallo et al., 2011; Sukasem et al., 2014; Su et al., 2016; Sukasem et al., 2016)	
	HLA-C*03:02	SJS/TEN	7.77	2.78	0.38	1.07	0.27	7.66	(Cristallo et al., 2011; Li et al., 2017)	
	HLA-A*33:03	SJS/TEN	11.17	3.97	0.57	1.07	0.53	11.70	(Cristallo et al., 2011; Li et al., 2017)	
	HLA-C*08:01	SJS/TEN	10.32	0.20	0.00	1.71	2.41	11.28	(Cristallo et al., 2011)	
Abacavir	HLA-B*57:01	AHS	1.17	2.39	4.15	1.92	2.14	0.97	(Sukasem et al., 2014; Dao et al., 2015; Su et al., 2016)	
Nevirapine	HLA-B*35:05	SJS/TEN, DRESS	1.91	0.00	0.38	0.85	0.00	0.14	(Chantarangsu et al., 2009; Sukasem et al., 2014; Lauren et al., 2014)	
Co-trimoxazole	HLA-B*38:01	SJS/TEN	0.00	0.40	2.45	1.71	1.07	0.42	(Lonjou et al., 2008; White et al., 2014)	
	HLA-B*38:02	SJS/TEN	4.26	0.00	0.19	0.00	0.00	6.55	(Lonjou et al., 2008; White et al., 2014)	
	HLA-B*15:02	SJS/TEN	7.66	0.20	0.00	0.00	0.00	4.87	(Kongpan et al., 2015)	
	HLA-C*06:02	SJS/TEN	4.26	11.31	8.68	6.84	5.62	3.62	(Kongpan et al., 2015)	
	HLA-C*08:01	SJS/TEN	10.32	0.20	0.00	1.71	2.41	11.28	(Kongpan et al., 2015)	
Dapsone	HLA-B*13:01	DRESS	5.96	0.00	0.00	0.00	0.00	3.34	(Zhang et al., 2013; White et al., 2014; Tempark et al., 2017)	
Salazosulfa- Pyridine	HLA-B*13:01	DRESS	5.96	0.00	0.00	0.00	0.00	3.34	(Yang et al., 2014)	
Methazolamide	HLA-B*59:01	SJS/TEN	0.00	0.00	0.00	0.00	0.00	0.56	(Stephens et al., 2013; White et al., 2014; Yang et al., 2016)	
Amoxicillin– Clavulanate	HLA-A*30:02	DILI	0.00	4.96	0.57	3.42	1.87	0.14	(Stephens et al., 2013; White et al., 2014; Yang et al., 2016)
Ticlopidine	HLA-A*33:03	DILI	11.17	3.97	0.57	1.07	0.53	11.70	(Hirata et al., 2008)
Flucloxacillin	HLA-B*57:01	DILI	1.17	2.39	4.15	1.92	2.14	0.97	(Daly et al., 2009)

HLA-A, human leukocyte antigen-A; HLA-B, human leukocyte antigen-B; HLA-C, human leukocyte antigen-C; AHS, abacavir hypersensitivity; CADRs, cutaneous adverse drug reactions; DRESS, drug reactions with eosinophilia and systemic symptoms; DILI, drug-induced liver injury; MPE, maculopapular exanthema; SCARs, severe cutaneous adverse reactions; SJS, Stevens–Johnson syndrome; TEN, toxic epidermal necrolysis.

HLA-B*58:01 (IMGT/HLA ID: HLA00386) allele was similarly distributed in Thais (6.38%), African Americans (6.37%), and Asians (7.38%) (Yang et al., 2016) and higher than those reported in Caucasians (Cao et al., 2001), Hispanics (Cao et al., 2001), and North Americans (Cao et al., 2001) (**Tables 7**, **8A**). However, HLA-B*58:01 allele was not significantly different across different populations. Furthermore, other *HLA* alleles which are associated with allopurinol-induced SJS/TEN such as HLA-A*33:03 (IMGT/HLA ID: HLA00106), HLA-C*03:02 (IMGT/HLA ID: HLA00410), and HLA-DRB1*15:02 (IMGT/HLA ID: HLA00867) alleles were found to have higher frequency in Thai population than others. Note that the high distribution of HLA-DRB1*13:02 (IMGT/HLA ID: HLA00798) allele was observed in African Americans (8.5%) and Japanese (7.7%) but not so much (1.38%) among Thais (**Tables 8A**, **B**).

HLA-B*13:01 (IMGT/HLA ID: HLA00152) allele has been reported to be associated with dapsone and salazosulfapyridine-induced drug reaction with eosinophilia and systemic symptoms (DRESS). The frequencies of HLA-B*13:01 were similar among Thai population (*p*-value = 0.7450) and higher than African Americans, Caucasians, Hispanics, and North American (**Tables 7**, **8A**). Moreover, co-trimoxazole-induced SJS/TEN associated with HLA-B*15:02 (7.66%) and -C*08:01 (10.32%) alleles was higher within Thai population than that of African Americans, Caucasians, Hispanics, and North American. Furthermore, HLA-A*33:03 allele which is associated with ticlopidine-induced liver injury appeared to be higher (11.17%) than the allele frequencies from Caucasians (Cao et al., 2001), Hispanics (Cao et al., 2001), and North American (Cao et al., 2001). The allele frequencies of HLA-DQB1*06:02 (IMGT/HLA ID: HLA00646) allele which is associated with amoxicillin–clavulanate-induced liver injury were found to have higher frequency in African Americans and Caucasians in contrast to the allele frequency from Thai population (**Table 8B**).

**Table 8B:** Comparison of pharmacogenetics markers in Thai and other populations.

Drug	Pharmacogenetics markers	ADR type	Allele frequency (%)					Reference
			Thai population (n = 470)	African Americans (n = 241) (Just et al., 1996)	Caucasians (n = 265) (Begovich et al., 1992)	Japanese (n = 371) (Saito et al., 2000)	Han Chinese (n = 10,689) (Zhou et al., 2016)	
Carbamazepine	HLA-DRB1*12:02	SJS/TEN	15.32	0.40	0.00	1.50	7.16	(Shi et al., 2017)
Phenytoin	HLA-DRB1*16:02	SJS/TEN	5.96	0.00	0.00	0.90	2.61	(Hung et al., 2010; White et al., 2014)
Allopurinol	HLA-DRB1*13:02	SJS/TEN	1.38	8.50	3.40	7.70	3.54	(Cristallo et al., 2011)
	HLA-DRB1*15:02	SJS/TEN	14.47	0.00	0.80	10.00	2.91	(Cristallo et al., 2011)
Amoxicillin– Clavulanate	HLA-DRB1*15:01	DILI	8.09	16.00	15.80	8.50	12.74	(Lucena et al., 2011; Stephens et al., 2013)
	HLA-DQB1*06:02	DILI	1.49	23.20	15.80	8.20	10.69	(Lucena et al., 2011; Stephens et al., 2013)
Lapatinib	HLA-DQA1*02:01	DILI	8.72	9.10	13.20	N/A	7.28	(Spraggs et al., 2011)

HLA-DRB1, human leukocyte antigen-DRB1; HLA-DQA1, human leukocyte antigen-DQA1; HLA-DQB1, human leukocyte antigen-DQB1; DILI, drug-induced liver injury; SJS, Stevens–Johnson syndrome; TEN, toxic epidermal necrolysis; N/A, not available.

### Population Structure Analysis

**Figure 2:**
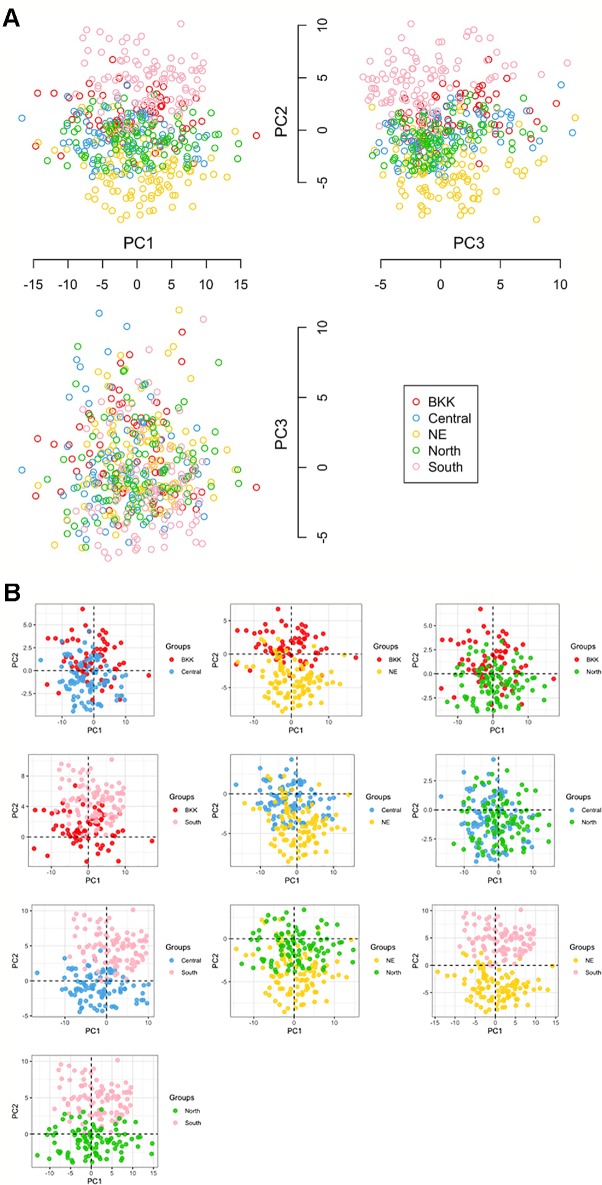
**(A)** Principal component analysis (PCA) analysis in ggplot library and KRIS library with z-score as the input [five groups: 100 North, 100 NE (Northeastern), 70 BKK (Bangkok), 100 Central, and 100 South]. **(B)** All compare between each population by Principal Component Analysis (PCA).

We used PCA to observe potential subgroups within a given 470 Thai individuals. Instead of using only six *HLA* halplotypes (from both class I and II), we extract 403 polymorphism probes and used them in the analysis. PCA revealed three prominent subpopulations in which samples from Northeastern (NE:yellow) and Southern (South:pink) groups were somewhat separated (see plots of PC1 vs. PC2 and PC2 vs. PC3 in **Figure 2**). The third subpopulation—placed in the middle of Northeastern and Southern groups—comprised samples from Central (sky blue), North (green), and Bangkok (BKK:red). The PCA plot between PC1 vs. PC3 did not clearly show subpopulations.

**Figure 3:**
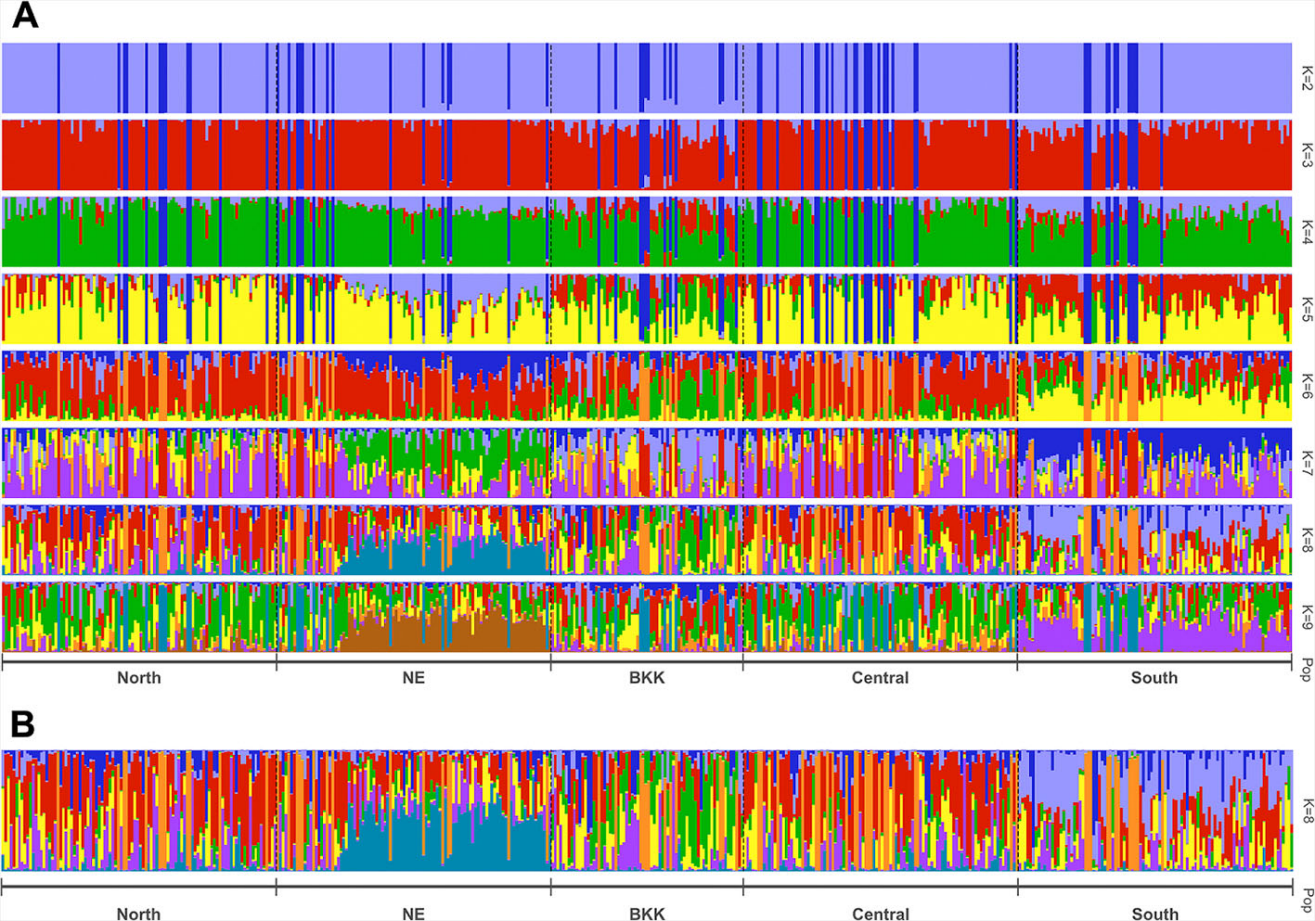
Estimated population structure with z-score as the input based on STRUCTURE analysis with pophelper visualization tool. Bar plot of individual ancestry proportions for the genetic clusters inferred using **(A)** K = 2 to 9, and **(B)** optimal K = 8.

STRUCTURE uses Bayesian to infer/predict a contribution to potential subpopulations (K) from estimated genetic variation frequencies. In our case, 403 *HLA* polymorphism probe signals were used to represent the genetic variations. Since this work recruited volunteers from five regions based on recent demographic information, we set K = 10 to cover these five demographic groups. CLUMPAK reported eight as the best number of genetic groups (subpopulations) for both Evanno’s (Δ K) and Pritchard’s (likelihood K). **Figure 3** shows patterns of individuals’ assignments to K populations. Each row in this figure shows proportion of individuals’ contribution to K subpopulations, from K = 2 to K = 9. In particular, K colors on each vertical bar reveal genetic composition (admixture). **Figure 3** shows the admixture profiles for eight genetic groups (K = 8). All 470 individuals are represented by vertical bars, which are grouped according to their reported five geographical origins. Two distinct admixture patterns of Northeastern and Southern regional groups could be observed, while Bangkok and Central regional groups’ admixture patterns tend to be similar.

## Discussion

In this study, we examined the frequency distribution of *HLA* class I and II alleles in five regions of Thailand by *HLA*-typing of 470 healthy individuals using PCR-SSOs probe. We found the most prevalent alleles of *HLA* class I and II were HLA-A*11:01, -B*46:01, -C* 01:02, -DRB1*12:02, -DQA1*01:01, and -DQB1*05:02. When compared with the previous study, the allele frequency of *HLA-B,* our frequency report from 470 healthy cohort was similar to the previous one (Puangpetch et al., 2015). Especially, pharmacogenetic markers associated with different ethnic groups such as HLA-A*33:03, -B*15:02, -B*13:01, -C*03:02, -DRB1*12:02, -DRB1*15:02, -DRB1*16:02, and -DQB1*06:02.

Recent studies confirm that SCARs are caused by certain *HLA* polymorphisms, drugs, peptide, and T cell (Yun et al., 2012; Iasella et al., 2017). There are four phenotypically distinct SCARs including SJS, TEN, DRESS, or drug-induced hypersensitivity syndrome (DIHS) or hypersensitivity syndrome (HSS), and acute generalized exanthematous pustolosis (AGEP) (Marotti, 2012). SJS and TEN are severe life-threatening reactions and are associated with ~5% mortality rate, >30% SJS and >30% TEN (Aihara, 2011). The incidence of SJS/TEN in Southeast Asia (Brunei, Cambodia, Indonesia, Laos, Malaysia, Myanmar, Philippines, Singapore, Vietnam, and Thailand) is largely undetermined. Nevertheless, more and more incidences of culprit drugs have been published, including CBZ (17%), allopurinol (15%), beta-lactam antibiotics (13%), sulfonamide antibiotics (12%), PHT (9%), nonsteroidal anti-inflammatory drugs (8%), LTG (2%), and phenobarbital (1%) (Lee et al., 2013). According to the data from the spontaneous reporting of adverse drug reactions during 1984–2014 by Thailand Health Product and Vigilance Center (HPVC), the culprit drugs causing SJS and TEN in Thailand include sulfamethoxazole and trimethoprim, allopurinol, CBZ, nevirapine containing produce, PHT, amoxicillin, phenobarbital, ibuprofen, tetracycline, piroxicam, diclofenac, rifampicin, ceftriaxone, fluconazole, isoniazid, ciprofloxacin, ethambutol, pyrazinamide, amoxicillin and clavulanic acid, and dapsone. (http://thaihpvc.fda.moph.go.th/thaihvc/Public/News/uploads/hpvc_1_3_4_100538.pdf). DRESS is characterized by fever, cutaneous eruption, internal organ involvement (hepatitis), and hematologic abnormalities (eosinophilia and/or atypical lymphocytosis). The onset of DRESS is approximately 1–8 weeks after drug initiation with the mortality rate of approximately 10% (Criado et al., 2012). From 2004 to 2014, the King Chulalongkorn Memorial Hospital Thailand reported list of drugs that could cause DRESS in Thai patients, including PHT, nevirapine, allopurinol, co-trimoxazole, dapsone, CBZ, isoniazid, ciprofloxacin, clindamycin, dacomitinib, danazol, omeprazole, and sulfadiazine (Hiransuthikul et al., 2016).

Abacavir is used for treatment of human immunodeficiency virus type I infection (Sukasem et al., 2014; White et al., 2014). Abacavir-hypersensitivity reaction (ABC-HSR) is a major of adverse drug reactions, and its reaction onset occurs during the first 6 weeks of treatment (Chaponda and Pirmohamed, 2011). Previous studies showed the association of HLA-B*57:01 allele with ABC-HSR in European and African (Mallal et al., 2008; Sukasem et al., 2014; White et al., 2014). The data from 470 Thais showed that the allele frequency of HLA-B*57:01 was not so common (1.17% of 470): 1.4% Bangkok, 1.5% Central, 1% Northeastern, 1% Northern, and 1% Southern. Our results confirmed the previous study of 986 Thais which revealed 1.5% of HLA-B*57:01 allele distribution in Thailand (Puangpetch et al., 2015). The range of HLA-B*57:01 allele distribution was more common (0.97%–4.15%) in African Americans, Caucasians, Hispanics, North American, and Asians (Cao et al., 2001). The distribution of Thai HLA-B*57:01 allele was closer to Southeast Asian populations: 2.9% of 170 Vietnamese and 1.27% of 237 Indonesian (Hoa et al., 2008; Yuliwulandari et al., 2009). Thus, the allele frequency of HLA-B*57:01 has confirmed the value of the screening among different ethnicities before initiation of treatment.

CBZ-induced SJS/TEN was strongly associated with HLA-B*15:02 in Han Chinese, Thais, Vietnamese, Malaysian, and Indian (Ferrell and McLeod, 2008; Locharernkul et al., 2008; Hung et al., 2010; Chang et al., 2011; Nguyen et al., 2015). We found the allele frequency of HLA-B*15:02 was 0.0766 in Thais, especially, among individuals from Southern Thailand. Particularly, the high expression of HLA-B75 family distribution (HLA-B*15:02, -B*15:11, and HLA-B*15:21) in the Southern group was a genetic factor associated with CBZ-induced SJS/TEN. Nevertheless, the distribution of HLA-B*15:02 allele in Caucasian and Japanese populations is less than 1% and African Americans is 0.20% (Amstutz et al., 2014; White et al., 2014). Therefore, HLA-B*15:02 allele is quite ethnic specific as well as having phenotype of SCARs. Furthermore, other studies supported HLA-A*31:01 associated with CBZ-induced SJS/TEN, DRESS, and maculopapular exanthema (MPE) in European, Japanese, Taiwan Han Chinese, and Korean (McCormack et al., 2011; Ozeki et al., 2011; Amstutz et al., 2014). The allele frequency of HLA-A*31:01 was higher among North Americans (7.75%), Hispanics (4.91%), and Asians (3.06%), while Thais’ and African Americans’ were much lower, 0.85% and 0.79%, respectively (Cao et al., 2001). The report also shows that HLA-A*31:01 is more common in most ethnic groups (Amstutz et al., 2014). The discordant allele frequencies in which Thais harbor less common HLA-A*31:01 allele supports the need to study more ethnic-specific *HLA* alleles among Southeast Asian populations. Additionally, the cross-reactivity between PHT-induced SJS/TEN and OXC-induced SJS/TEN was associated with HLA-B*15:02 among Han Chinese (Hu et al., 2011; Su et al., 2016). Previous studies showed that CBZ-, PHT-, and LTG-induced SJS/TEN and MPE were associated with HLA-A*24:02 (Moon et al., 2015; Shi et al., 2017). We found the distribution of HLA-A*24:02 allele was 0.1149 in Thailand which was also with similar rate across all five specific regions in Thailand. Although HLA-A*24:02 allele frequency in African Americans was much lower (0.0278), this *HLA* allele was used as a predictive marker for antiepileptic drug-induced SJS/TEN and MPE (Cao et al., 2001). The frequency distributions of pharmacogenetics markers from the neighboring countries were quite similar to Thais’, including Vietnamese: HLA-B*15:02 (0.135) and HLA-A*24:02 (0.138), Indonesian: HLA-B*15:02 (0.116) and HLA-A*24:02 (0.143), Myanmar: HLA-B*15:02 (0.088) and HLA-A*24:02 (0.168), and Malaysian: HLA-B*15:02 (0.0825) (Hoa et al., 2008; Yuliwulandari et al., 2009; Jinam et al., 2010; Kongmaroeng et al., 2015). Similarly, the association between frequency of specific *HLA* alleles and antiepileptic drug-induced SCARs was investigated in many populations.

Allopurinol-induced SJS/TEN causes disease spectrum, including skin rashes, fever, vasculitis, hepatitis, and epidermal necrosis (Lupton and Odom, 1979; Halevy et al., 2008; Tassaneeyakul et al., 2009). For Thai patients, HLA-B*58:01 is strongly associated with allopurinol-induced SJS/TEN (odds ratio = 579.0; 95% CI: 29.5–11362.7; p-value < 0.001), DRESS (odds ratio = 430.3; 95% CI: 22.6–8958.9; p-value < 0.001), and MPE (odds ratio = 144.0; 95% CI: 13.9–1497.0; p-value < 0.001) (Sukasem et al., 2016). HLA-B*58:01 was reported to be present 8%–15% among Han Chinese and Thai population, 0.6% of Japanese, and 0.8% of European population (Hung et al., 2005; Kaniwa et al., 2008; Tassaneeyakul et al., 2009; Somkrua et al., 2011). In this study, the frequency of HLA-B*58:01 allele was 0.0638 in 470 Thai population, ranging between 0.0500 and 0.0800 when considering at the regional group level. Note that the frequency of HLA-B*58:01 allele in Thailand was not much different when compared with other populations such as African Americans, Caucasians, Hispanics, North American, Asians, and Southeast Asians (Malaysia, Vietnam, Indonesia, and Myanmar) (Cao et al., 2001; Hoa et al., 2008; Yuliwulandari et al., 2009; Jinam et al., 2010; Kongmaroeng et al., 2015). Therefore, it is likely that the distribution of HLA-B*58:01 could be used as a universal pharmacogenetic marker for allopurinol-induced CADRs including SJS/TEN, DRESS, and MPE for all ethnicities.

Reports showed the association among Han Chinese leprosy patients and Thai non-leprosy patients between HLA-B*13:01 and dapsone hypersensitivity reactions (odds ratio 122.1, p-value = 6.038 10^−12^ and odds ratio 20.53, p-value = 6.84 10^−25^) and dapsone-induced SCARs (odds ratio 54.00, p-value = 0.0001) and dapsone-induced DRESS (odds ratio 60.75, p-value = 0.0001), respectively (Wang et al., 2013; Zhang et al., 2013; Tempark et al., 2017). The distribution of HLA-B*13:01 allele was absent in Europeans and Africans (Cao et al., 2001; Zhang et al., 2013). Recent publications showed varying distributions of HLA-B*13:01, including 1.5% of Japanese, 1%–12% of Indian, 28% of Papuans and Australian aborigines, 2%–20% of Chinese, 2%–4% of Southeast Asians, 1.94% of Koreans, and 6.95% of the previously reported Thai population (Cao et al., 2001; Zhang et al., 2013; Puangpetch et al., 2015; Tempark et al., 2017). This study showed that the frequency of HLA-B*13:01 allele was 0.0596 which is similar to the previous study in Thailand (Puangpetch et al., 2015). Furthermore, the frequency of HLA-B*13:01 allele was shown to have a very strong association with many drug-induced DRESS (PHT, phenobarbital, dapsone, and salazosulfapyridine) and Asian populations (Thais, Han Chinese, Malaysian, Vietnamese, Indonesian, and Myanmarese) (Hoa et al., 2008; Yuliwulandari et al., 2009; Jinam et al., 2010; Kongmaroeng et al., 2015). Interestingly, dapsone shows similarity in chemical structure to the sulfonamides; the cross-reactivity of sulfonamide hypersensitivity reactions was also reported (Tomecki and Catalano, 1981). Co-trimoxazole (sulfonamide and trimethoprim) is a sulfonamide antibiotic and the most common culprit drug for SJS/TEN in many countries including Thailand (Barvaliya et al., 2011; Kongpan et al., 2015). The study reported the association of the alleles HLA-B*38:01 and HLA-B*38:02 with sulfamethoxazole-induced SJS/TEN in European patients (Lonjou et al., 2008). Furthermore, HLA-B*15:02 allele was found to be associated with co-trimoxazole-induced SJS/TEN in Thais (odds ratio 3.91, p-value = 0.0037) (Kongpan et al., 2015). In this study, we found the frequencies of HLA-B*38:01 allele was 0.000, HLA–B*38:02 allele was 0.043, and HLA-B*15:02 allele was 0.077. With the presence of these alleles, further investigation should be conducted on other *HLA* alleles and co-trimoxazole-induced SCARs in Thai population.

Drug-induced liver injury (DILI) is rare, but it potentially causes serious idiosyncratic reaction (Kaplowitz, 2005). Previous study reported the association between amoxicillin–clavulanate-induced liver injury and *HLA* haplotypes: HLA-DRB1*15:01, HLA-DQB1*06:02, and HLA-A*30:02 (Stephens et al., 2013). In this study, we found that the frequencies of HLA-DRB1*15:01 and *HLA*-DQB1*06:02 were 0.0809 and 0.0149, respectively. However, HLA-A*30:02 allele was absent in Thai population. We noticed that the distribution of HLA-DQB1*06:02 allele in Thai population was much lower than those found in African Americans and Caucasians (Begovich et al., 1992; Just et al., 1996). The distribution of HLA-DQB1*06:02 was quite similar among Southeast Asian countries (Malaysia, Vietnam, and Myanmar) and was similar to Thais (Hoa et al., 2008; Jinam et al., 2010; Kongmaroeng et al., 2015). Additionally, the frequency of a pharmacogenetic marker associated with ticlopidine-induced hepatotoxicity, HLA-A*33:03, was 0.1117 reported by our *HLA* study of Thai cohort. Similarly, the frequencies of this pharmacogenetics marker were 0.1150 for Vietnamese, 0.1516 for Indonesian, 0.1380 for Burmese, and 0.08 for Malaysian (Hoa et al., 2008; Yuliwulandari et al., 2009; Jinam et al., 2010; Kongmaroeng et al., 2015). According to trends of *HLA* prevalence of Southeast Asian, both HLA-A*33:03 and HLA-DQB1*06:02 allele should be further investigated in which they can be used as pharmacogenetic markers in Thais and other neighboring populations.

Previous report demonstrated that Thai population contains four prominent substructures using 435,503 single-nucleotide polymorphisms (SNPs) collected from two independent studies comprising 992 Thai individuals (Wangkumhang et al., 2013). The analysis from their work revealed two important concepts: 1) the three main subpopulations are localized in Northern, Northeastern, and Southern parts of Thailand while the members from the fourth group are scattered throughout the country and 2) place of origins of Thai individuals might be discordant with the genetic similarity profile of that place. In other words, people from the north could migrate to the south and stay there for more than three generations. Similar findings were shown in our population genetic analyses in which Northeastern and Southern groups were genetically different while Bangkok and Central groups were mixed and/or scattered to Northern, Northeastern, and Southern groups. In terms of *HLA*-haplotype distributions over the five regions, we found that the haplotype frequencies in these five regions were slightly different. However, we did not observe novel HLA-haplotypes specific to any subregion. The PCR probes used to call these *HLA*-CLASS I and CLASS II rely on some known collections of *HLA*s and might not be able to predict novel *HLA* haplotypes.

Ethnic-specific genetic variation database is vital to identification of good pharmacogenetic markers in Asian countries such as CYP2C9*3 associated with PHT-induced SJS/TEN in Taiwanese, Japanese, and Malaysians (Chung et al., 2014). Further studies should be done to confirm pharmacogenetics markers from the ethnic-specific SNP databases. Since there were only six haplotypes and 470 individuals, the challenge of this study was the data analyses obtained from the platform called PCR-SSO probe. To address the lack of genetic polymorphisms in our population genetic study, we extracted probe signals laid across six stretches of *HLA*. Using the probe signals, we observed some distinct as well as cline subpopulations. This discrepancy should be clarified in further study by performing high-resolution DNA typing and recruiting more Thai individuals. In this study, we identified both *HLA* class I and II alleles in Thai population. Furthermore, many *HLA* class I and II alleles were associated with pharmacogenetics markers which might appear exclusively in many populations. Particularly, a database containing distribution of specific *HLA* class I and II alleles will support the development of the pharmacogenetics markers for screening drug hypersensitivity reactions.

## Data Availability Statement

The datasets used in this study can be found here https://www.ebi.ac.uk/ipd/imgt/hla using the accession numbers HLA-A*31:01 (IMGT/HLA ID: HLA00097), HLA-A*33:03 (IMGT/HLA ID: HLA00106), HLA-B*13:01 (IMGT/HLA ID: HLA00152), HLA-B*15:02 (IMGT/HLA ID: HLA00165), HLA-B∗15:08 (IMGT/HLA ID: HLA00171), HLA-B∗15:11 (IMGT/HLA ID: HLA00174), HLA-B∗15:21 (IMGT/HLA ID: HLA00184), HLA-B*35:05 (IMGT/HLA ID: HLA00241), HLA-B*57:01(IMGT/HLA ID: HLA00381), HLA-B*58:01(IMGT/HLA ID: HLA00386), HLA-B*59:01 (IMGT/HLA ID: HLA00389), HLA-C*03:02 (IMGT/HLA ID: HLA00410), HLA-C*06:02 (IMGT/HLA ID: HLA00430), HLA-C*08:01 (IMGT/HLA ID: HLA00445), HLA-DRB1*13:02 (IMGT/HLA ID: HLA00798), HLA-DRB1*15:02 (IMGT/HLA ID: HLA00867) and HLA-DQB1*06:02 (IMGT/HLA ID: HLA00646).

## Ethics Statement

The studies involving human participants were reviewed and approved by the Ethical Clearance Committee on Human Rights Related to Research Involving Human Subjects Faculty of Medicine Ramathibodi Hospital Mahidol University. The patients/participants provided their written informed consent to participate in this study.

## Author Contributions

PS had substantial contributions to the conception, design, analysis, and interpretation of the data, drafting the manuscript, and agrees to be accountable for all aspects of the work. PJ, TJ, NK, CC, JP, CNa, WA, ST, CN and AW had substantial contributions to the conception and analysis of the data and drafting the manuscript. CS had substantial contributions to the conception and design of the work, drafting the work, and agrees to be accountable for all aspects of the work in ensuring that questions related to the accuracy or integrity of any part of the work are appropriately investigated and resolved and provide approval for publication of the content.

## Conflict of Interest

The authors declare that the research was conducted in the absence of any commercial or financial relationships that could be construed as a potential conflict of interest.

The reviewer TM declared a past co-authorship with one of the authors CS to the handling editor.
